# Temporal dynamics in mental health symptoms and loneliness during the COVID-19 pandemic in a longitudinal probability sample: a network analysis

**DOI:** 10.1038/s41398-023-02444-z

**Published:** 2023-05-10

**Authors:** Michael Odenthal, Pascal Schlechter, Christoph Benke, Christiane A. Pané-Farré

**Affiliations:** 1 University of Marburg, Marburg, Germany; 2 University of Cambridge, Cambridge, UK

**Keywords:** Pathogenesis, Human behaviour

## Abstract

Figuring out which symptoms are central for symptom escalation during the COVID-19 pandemic is important for targeting prevention and intervention. Previous studies have contributed to the understanding of the course of psychological distress during the pandemic, but less is known about key symptoms of psychological distress over time. Going beyond a pathogenetic pathway perspective, we applied the network approach to psychopathology to examine how psychological distress unfolds in a period of *maximum stress* (pre-pandemic to pandemic onset) and a period of *repeated stress* (pandemic peak to pandemic peak). We conducted secondary data analyses with the Understanding Society data (*N* = 17,761), a longitudinal probability study in the UK with data before (2019), at the onset of (April 2020), and during the COVID-19 pandemic (November 2020 & January 2021). Using the General Health Questionnaire and one loneliness item, we computed three temporal cross-lagged panel network models to analyze psychological distress over time. Specifically, we computed (1) a *pre-COVID to first incidence peak network*, (2) a *first incidence peak to second incidence peak network*, and (3) a *second incidence peak to third incidence peak network*. All networks were highly consistent over time. *Loneliness* and *thinking of self as worthless* displayed a high influence on other symptoms. *Feeling depressed* and *not overcoming difficulties* had many incoming connections, thus constituting an end-product of symptom cascades. Our findings highlight the importance of loneliness and self-worth for psychological distress during COVID-19, which may have important implications in therapy and prevention. Prevention and intervention measures are discussed, as single session interventions are available that specifically target loneliness and worthlessness to alleviate mental health problems.

## Introduction

The coronavirus disease 2019 (COVID-19) pandemic and related government-imposed lockdown measures have affected social life and mental health worldwide [1, 2]. In large prospective-longitudinal studies including the Understanding Society Study, a nationally representative, longitudinal household study from the UK, it has been demonstrated that (a) nearly 40% of the examined population experienced a significant increase in psychological distress during the pandemic, (b) psychological distress peaked during all lockdown periods with its maximum in the third lockdown and (c) mental health problems decreased during easing of lockdown restrictions [3–7]. This pattern has been observed consistently across countries [8–13].

The existing studies have helped to identify different mental health trajectories and psychological or sociodemographic factors (e.g., being female, young, lonely) related to the deterioration of mental health during the pandemic (e.g, ref. [6]). However, the studies typically obtained composite scores to examine interindividual differences or intraindividual changes in mental health which can obscure the identification of important subtleties in dynamic symptom changes and interactions between symptoms over time. For instance, the importance of single symptoms like enjoying day-to-day activities, loneliness, the ability to concentrate, sleeping problems, decision-making problems, self-confidence, or happiness and their interaction in light of governmental restrictions remain masked. Moreover, investigating temporal dynamics of complex symptom networks is critical to identify central symptoms responsible for the escalation of other symptoms over time, e.g., during the COVID-19 pandemic, which, then, can be potentially targeted in intervention and prevention [14].

To elucidate the complex and dynamic interactions among symptoms, network analytical approaches have been proposed [15]. From the perspective of the network approach to psychopathology [16, 17] co-occurring psychopathological symptoms, referred to as nodes, are connected with each other to a greater or lesser extent (these connections between symptoms are also referred to as edges). Thus, it is assumed that symptoms mutually influence and possibly reinforce each other, with central symptoms characterized by a substantial influence on other symptoms. These symptoms may prove clinically relevant because, in theory, they lead to an activation of the entire network, thus increasing symptom activation [18].

In the context of the COVID-19 research, loneliness has been identified as a central relevant symptom. In repeated cross-sectional studies, there is first indication that loneliness was the most central variable in mental health networks (i.e., depression, anxiety, stress, and poor sleep [19]). In addition, the partial correlations (i.e., symptom connectivity in network analysis) between the affective symptoms and social isolation increased during lockdown, potentially indicating that socially isolated groups are more vulnerable to affective disorders in the face of this population stressor [20]. However, in a network analysis in an Irish sample, loneliness was not centrally related to anxiety and depression [21]. In addition, studies have revealed a central influence of worthlessness within mental health networks. Worthlessness exhibited the highest influence in a network of pandemic-related mental health conditions and symptoms of major depressive disorder and generalized anxiety disorder [22]. In addition, worthlessness functioned as bridge symptom in a cross-sectional network, exerting a high influence on the activation of the relationship between of depression and parental stress [23]. Loneliness and worthlessness appear to be particularly relevant to be studied in a longitudinal approach, as these cross-sectional studies provide a limited perspective and cannot unravel unfolding dynamic symptom changes, which is particularly relevant for prolonged stressors as pandemics. Accordingly, these studies do not distinguish whether a symptom was central because it had a strong influence on others or whether it was strongly influenced by others. In terms of symptom network activation, it is important to identify which symptoms lead to symptom activation as these may be central in intervention and prevention to counteract a symptom exacerbation at an early stage. Therefore, in line with the network theory, these symptoms may be viable targets to stop symptom activation in the network [15, 18, 24].

A longitudinal network analysis is clinically relevant as it enables a fine-grained identification of pre-COVID-19 symptoms that predispose symptom escalation during the lockdown as well as symptoms that emerge during COVID-19 and reinforce symptom manifestation. This may inform prevention and intervention programs at an early stage of network activation tailored to specific time-points in the pandemic. Only one study that used ecological momentary assessments compared the temporal network structure during a lockdown stage with the network structure of a no-lockdown stage in a German convenience sample (August 2020–March 2021), covering a “no-lockdown” and a “lockdown” stage [25]. In this study, loneliness centrally influenced other symptoms only during the lockdown but not before the lockdown. However, this study had a small sample, and did not examine how pre-COVID symptoms unfold and operate in a network during a lockdown. Network analyses that include data before the pandemic onset and at highly relevant time points across the entire pandemic (e.g., lockdowns), in particular disentangling ingoing and outgoing symptom connections, are currently lacking.

To close this research gap, we analyzed data from the Understanding Society Study, a representative prospective-longitudinal household study in adults from UK, using temporal network analysis. These networks can be used to identify dynamic symptom changes and, in particular, which symptoms have a central influence on the network at a subsequent time point. To pinpoint how symptoms unfold during a period of *maximum stress* for individuals, we examined dynamical change in symptom networks from the last pre-COVID survey (2019) to the first COVID wave in April 2020 (pre-COVID to first incidence peak network). To examine the effects of *repeated stress*, we therefore selected two further waves (November 2020, January 2021), as these time points can be seen as repeated stressors for individuals (a first incidence peak to second incidence peak network, and a second incidence peak to third incidence peak network). Based on prior literature, we expected that loneliness constitutes a symptom that is centrally influencing other symptoms [19, 20], thus triggering psychological distress symptom network activation. Given the lack of longitudinal studies, we exploratorily tested how symptoms like day-to-day activities, the ability to concentrate, sleeping problems, decision-making problems, self-confidence, or happiness operate and unfold in the networks without specifying further a priori hypotheses.

## Methods

### Participants and study design

We conducted a secondary data analysis by drawing on data from the *Understanding Society UK Household Longitudinal Study*, a nationally representative, prospective cohort study in the United Kingdom (UK) with about 40,000 households assessed since 2009 [26, 27]. Since April 2020, all members of the main Understanding Society samples aged 16 years and older in April 2020 were invited to participate, if they participated in at least one of the last two waves of data collection investigating various questions pertaining to individuals’ life’s during COVID-19 (*Understanding Society: COVID-19 Study)*. Until now, data for eight consecutive COVID-19 waves were collected in April, May, June, July, September, November 2020, as well as January 2021, and March 2021. All participants of the *Understanding Society: COVID-19 Study* subsample were assessed using online and telephone surveys.

In line with our research questions, we focused on the analysis of the most recent measurement occasion before the onset of the pandemic (2019/2020), the first time-point during the pandemic (April 2020, wave 1, *n* = 17,761), and the subsequent peaks, i.e., wave 6 (November 2020, *n* = 12,035) and wave 7 (January 2021, *n* = 11,968), as defined by COVID-19 incidence and stringency index (see supplement Fig. S18–S20 for the course of the incidence of COVID-19 cases, for confirmed deaths, and for the degree of the stringency of lockdown measures). In total, 17,761 participants participated in the first wave during the COVID-19 pandemic of the *Understanding Society: COVID-19* and were the target of the present analyses. Among the 34,318 participants who participated in the last assessment of the pre-COVID-19 survey (i.e. before the COVID-19 pandemic), the response to the first wave during COVID-19 was ~51,75%. This is consistent with the individual-level online response levels of the main study, which was 50–55%, which increased to 85–90% through face-to-face surveys [28]. Full demographic characteristics of our sample including the sample sizes for each sampling time are depicted in Table 1. Ethical approval was granted by the University of Essex Ethics Committee (ETH1920-1271). The data are openly available to researchers via the UK Data service (https://ukdataservice.ac.uk). Detailed information on the *Understanding Society UK Household Longitudinal Study and Understanding Society: COVID-19 Study*, including the sample structure, subsamples, and panel attrition, has been previously presented [6, 29]. All procedures and measures collected in the *Understanding Society Study* are described at https://www.understandingsociety.ac.uk/about/study-content. For our secondary data analysis additional ethical approval was not required. The authors assert that all procedures contributing to this work comply with the Helsinki Declaration of 1975, as revised in 2013.

**Table 1:** Demographic characteristics for each sampling time.

Total (%) / Mean (SD)	Pre-COVID survey	April 2020 (wave 1)	November 2020 (wave 6)	January 2021 (wave 7)
*N*	*N* = 34,318	*N* = 17,761	*N* = 12,035	*N* = 11,968
	December 2018 to January 2020	April 2020	November 2020	January 2021
**Sex**				
Male	15,383 (44.8%)	7411 (41.7%)	4992 (41.5%)	4928 (41.2%)
Female	18,935 (55.2%)	10,334 (58.2%)	7033 (58.4%)	7030 (58.7%)
**Age**	49.85 (18.87)	50.53 (17.06)	54.57 (16.13)	54.82 (16.12)
**Ethnicity**				
White	28,097 (81,9%)	15,012 (84.5%)	10,634 (88.4%)	10,572 (88.3%)
Non-White	6068 (17,7%)	2124 (11.9%)	1146 (9.5%)	1111 (9.3%)
**Financial**				
Comfortably	10,250 (29,9%)	5895 (33.2%)	3704 (30.8%)	–
Doing alright	13,482 (39,3%)	7166 (40.4%)	5562 (46.2%)	–
Just right	6884 (20,1%)	2644 (14.9%)	1917 (15.9%)	–
Quite difficult	1955 (5,7%)	655 (3.7%)	417 (3.5%)	–
Very difficult	764 (2,2%)	256 (1.4%)	157 (1.3%)	–
**Partner**				
Yes	21,187 (61,7%)	12,532 (70.5%)	8,451 (70.2%)	8381 (70.0%)
No	13,131 (38,3%)	5229 (29.4%)	3583 (29.7%)	3587 (30.0%)
**COVID-19 Risk**				
No risk	–	10,859 (61.1%)	6774 (56.3%)	6667 (55.7%)
Moderate	–	5681 (31.9%)	4452 (37.0%)	4533 (37.8%)
High	–	1098 (6.2%)	774 (6.4%)	739 (6.2%)

*Note*. COVID-19 Risk variable: NHS variable that assign people to a risk of a severe disease following COVID-19 infection based on different health conditions and treatment types.

### Questionnaire measures

#### General Health Questionnaire 12 (GHQ-12)

The General Health Questionnaire 12 (GHQ-12) is a commonly applied measurement tool assessing mental health symptoms during the past 2 weeks [30]. Participants responded to each of the twelve items on a four-point Likert scale ranging from 0 “*not at all*” to 3 “*completely*”. Negative items were recoded so that higher GHQ 12 scores indicate poorer symptom expression. Internal consistency was good (α = 0.90 − 0.92 for all sampling times). A cutoff score of >11 has been proposed as an indicator of mental distress, as established by Goldberg et al. [31], replicated and validated among others by Ruiz et al. [32]. During the pre-COVID-19 survey, 37.86 % (*n* = 6725) suffered mental health deterioration above threshold, increasing to 50.08% (*n* = 8894) in wave 1, 53.43 % (*n* = 9489) in wave 6 and 53.95 % (*n* = 9582) in wave 7, based on the imputed sample.

#### Loneliness

Loneliness was assessed with a single item from the Government Statistical Service (GSS) harmonized principle of loneliness (https://gss.civilservice.gov.uk/guidances/harmonized-standards-guidance/); see also ref. [33]. Before COVID-19, the item read: “How often do you feel lonely?”. For the eight waves during COVID-19, the wording was: “In the last 4 weeks, how often did you feel lonely?”. In all sampling times (pre-COVID-19 and during COVID-19), the item was assessed on a 3-point Likert scale ranging from 1 = “*Hardly ever or never*”, 2 = “*Some of the time*” to 3 = “*Often*”.

### Missingness analysis and data imputation

Sample characteristics of the participants who dropped out of the study between April 2020 and January 2021 compared to those who stayed have been described elsewhere (see supplemental material in refs. [6, 29]). Briefly, characteristics that were associated with missingness were ethnicity (non-white had higher values of missingness than white participants), sex (males had higher levels of missingness than females), and age (older participants displayed higher levels of missingness than younger participants). Data were thus treated as missing at random (MAR) [34]. Excluding participants that did not complete all relevant questions would result in a final sample of *N* = 7815 complete cases. Thus, missing data were imputed. As multiple imputations [35] are not compatible with the present network analytical approach, we used one imputed dataset as recommended under such circumstances [36, 37]. To this end, we imputed the data with the MICE package in R [38] by using predictive mean matching [34]. In our imputation model, demographic characteristics that were associated with missingness, i.e. sex and ethnicity, were included as auxiliary variables. We performed a sensitivity analysis using full-cases only (see Supplemental Figs. S1 and S2). The results essentially remained the same compared to the imputed data set, increasing confidence in our results and their interpretation.

### Data analysis

We performed all analyses with the R Version 4.0.3 [39]. The R code to reproduce the current results is openly available on the OSF (https://osf.io/cusf3/?view_only=4edd973d5da24a20bbc2895a8288e2bd). We used cross-lagged panel network models (CLPN) to analyze the unfolding networks over time [37]. We analyzed three longitudinal networks: (1) *pre-COVID survey to wave 1*, (2) *wave 1 to wave 6*, (3) *wave 6 to wave 7*. In a first step, we calculated the regression coefficients for the models: We computed autoregressive pathways, where a symptom at one time-point predicts itself at the next time-point while adjusting for all other symptoms. Then, we computed cross-lagged pathways, where a symptom predicts another symptom while adjusting for the autoregressive effects and all other symptoms. For the estimation of the regression coefficients, we applied the least absolute shrinkage and selection operator (LASSO), which applies a penalization to avoid estimating spurious edges [40, 41]. We calculated our directional CLPNs from pre-measurement to first incidence peak and incidence peak to incidence peak using the *glmnet* package [37, 42]. The *qgraph* package [43] was used for the visualization of the networks using an average layout for the three different networks over time.

To quantify the centrality of symptoms in our directed CLPNs, we examined two centrality indices: in- and out-expected-influence. These indices are calculated by all outgoing symptom associations one respective item has with all other items in the following wave. This centrality measure is coined out-expected-influence (the degree to which a symptom predicts other symptoms). The other centrality metric is quantified by the ingoing symptom associations one item has with all other items and is labeled in-expected-influence (the degree to which a symptom is predicted by other symptoms). The edge weight difference test and centrality difference tests were used to determine possible significant differences in edges and centrality indices, respectively [44].

## Results

### Descriptive statistics

The GHQ items with the highest mean values (i.e., >1 in each sampling time) were *able to concentrate*, *playing a useful part*, *capable of making decisions*, *enjoy normal activities*, *can face up to problems*, and *feeling reasonably happy* (see Table 2). GHQ values increased from the pre-COVID-19 assessment compared to the first wave during COVID-19 in April 2020, *t*(15,464) = −11.22, *p* < .001, Hedges’s *g* = 0.185, indicating a deterioration of mental health. Comparing incidence peak to incidence peak, mental health problems in November 2020 (wave 6) were significantly higher than in April 2020 (wave 1), *t*(10,892) = −6.91, *p* < .001, Hedges’s *g* = 0.027 but January 2021 (wave 7) was not significantly different from November 2020 (wave 6), *t*(9,738) = −1.90, *p* = 0.058. Detailed descriptive statistics can be found in Table 2.

**Table 2:** Mean and standard derivation of each GHQ-12 and the loneliness item, sum scores for the GHQ-12 for each sampling time and internal consistencies (*N* = 7815).

	Pre-COVID survey	Wave 1	Wave 6	Wave 7
	Dec '18 to Jan ‘20	April 2020	November 2020	January 2021
GHQ items *mean* (SD)				
Able to concentrate	1.14 (0.45)	1.24 (0.62)	1.02 (0.52)	1.22 (0.54)
Lost much sleep	0.78 (0.7)	0.89 (0.82)	0.93 (0.74)	0.92 (0.75)
Playing a useful part	1.07 (0.48)	1.19 (0.71)	1.15 (0.52)	1.19 (0.56)
Capable of making decisions	1.05 (0.39)	1.09 (0.44)	1.10 (0.43)	1.12 (0.44)
Under stress	0.93 (0.75)	1.01 (0.81)	1.05 (0.76)	1.03 (0.78)
Could not overcome difficulties	0.70 (0.71)	0.72 (0.74)	0.82 (0.71)	0.81 (0.73)
Enjoy normal activities	1.14 (0.49)	1.50 (0.84)	1.42 (0.67)	1.48 (0.7)
Can face up to problems	1.07 (0.41)	1.09 (0.43)	1.11 (0.41)	1.12 (0.43)
Feeling unhappy and depressed	0.82 (0.79)	0.93 (0.85)	0.97 (0.81)	0.99 (0.82)
Losing confidence	0.70 (0.77)	0.69 (0.77)	0.79 (0.78)	0.77 (0.78)
Thinking of self as worthless	0.39 (0.67)	0.41 (0.69)	0.50 (0.71)	0.48 (0.71)
Feeling reasonably happy	1.08 (0.52)	1.17 (0.59)	1.19 (0.54)	1.24 (0.56)
Sum score GHQ (*SD*)	11.26 (5.48)	12.30 (6.08)	12.63 (6.01)	12.82 (6.12)
Cronbach’s alpha GHQ (α)	0.91	0.90	0.92	0.92
Omega total GHQ (Ω)	0.93	0.92	0.94	0.94
Loneliness	1.38 (0.60)	1.38 (0.60)	1.41 (0.59)	1.45 (0.61)
**Skewness/Kurtosis**				
Able to concentrate	1.67/4.64	0.86/1.19	1.41/2.83	1.37/2.36
Lost much sleep	0.63/0.27	0.58/-0.35	0.53/0.13	0.55/0.09
Playing a useful part	1.24/4.87	0.58/0.5	1.25/3.24	1.17/2.32
Capable of making decisions	1.49/7.85	1.4/5.52	1.75/6.18	1.93/6.35
Under stress	0.51/−0.02	0.44/−0.39	0.45/−0.03	0.49/−0.04
Could not overcome difficulties	0.81/0.39	0.81/0.32	0.63/0.35	0.71/0.45
Enjoy normal activities	1.56/4.35	0.23/−0.58	0.83/0.16	0.79/−0.16
Can face up to problems	1.76/7.96	1.78/6.67	2.27/7.68	2.25/7.33
Feeling unhappy and depressed	0.68/-0.12	0.51/−0.60	0.48/−0.37	0.44/−0.48
Losing confidence	0.87/0.20	0.96/0.42	0.79/0.21	0.82/0.22
Thinking of self as worthless	1.76/2.81	1.74/2.74	1.43/1.71	1.49/1.93
Feeling reasonably happy	1.01/3.56	0.76/1.53	1.24/2.54	1.16/1.82
Loneliness	1.30/0.62	1.33/0.71	1.10/0.21	1.01/−0.02

### Accuracy and stability

The bootstrapped confidence intervals around the edge weights are shown in Figs. S3–S5 (see supplemental material). The bootstrapped confidence intervals were small to moderate, indicating good accuracy of our networks. In addition, the case-drop bootstrapping results indicate a high stability of the centrality indices (Figs. S6– S8 in the supplemental material).

### Network comparison

The edge lists of all networks are presented in the supplemental materials *edg1*. The number of non-zero edges was consistent across networks [range: 125 (pre-COVID to first incidence peak) – 130 (second incidence peak to third incidence peak)]. The correlation of the edge lists between the networks was moderate to strong (range: *r* = 0.75 – *r* = 0.83). The overall correlation of the out-expected-influence (*r* = 0.89) and the overall correlation of the in-expected-influence (*r* = 0.84) between networks was high.

**Fig. 1:**
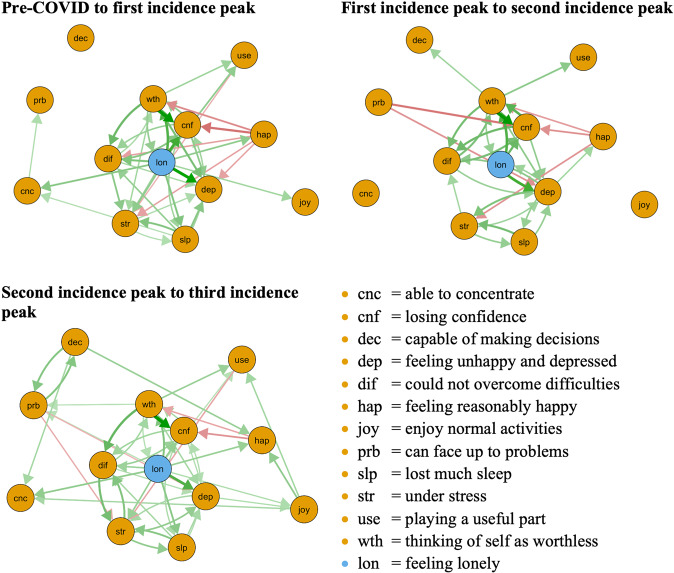
The cross-lagged panel networks for pre to incidence peak and incidence peak to incidence peak time-points. The relationship of the symptoms is indicated by the arrow’s color (green = positive, red = negative), the strength of the relationship is indicated by the arrow’s thickness (thicker = stronger). In these networks, autoregressive effects are excluded. Threshold was set to 0.05, excluding all relationships < 0.05. Final *N* = 17,761.

All three CLPN networks are depicted in Fig. 1: The symptoms *loneliness, feeling unhappy and depressed, thinking of self as worthless, losing confidence, could not overcome difficulties, under stress*, and *lost much sleep* formed a consistent cluster with strong connections over time. The symptoms *can face up to problems, capable of making decisions, feeling reasonably happy, able to concentrate*, and *enjoy normal activities* were less consistently connected across time.

**Fig. 2:**
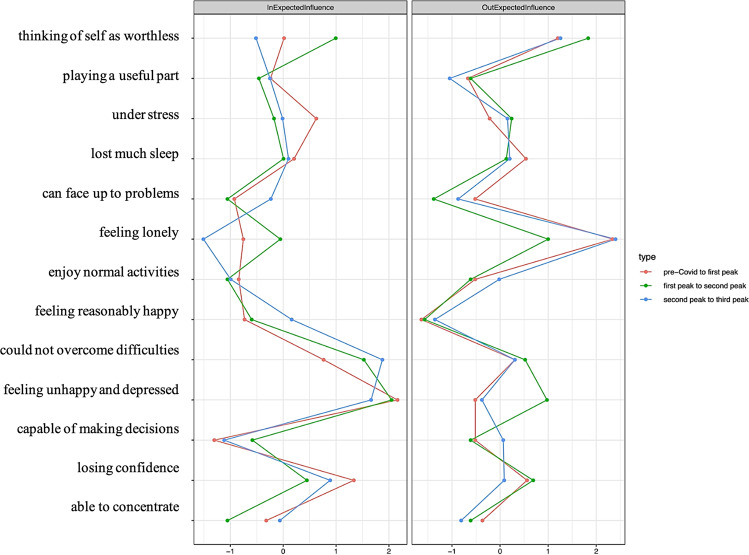
Symptom centrality estimates for the networks using z-values. Greater values indicate greater centrality. Out-expected-influence is the degree to which a symptom predicts other symptoms at the subsequent relevant point. In-expected-influence is the degree to which a symptom is predicted by other symptoms at the subsequent relevant point.

Table 3 depicts the five strongest edges for all three networks. Overall, the strongest edge connection in the three networks was the connection *thinking of self as worthless* → *losing confidence* (range: *β* = 0.15 – 0.20). In all three networks, the edge *loneliness* → *feeling unhappy and depressed* displayed the second strongest edge connection (range: *β* = 0.12 – 0.17). In the first network, the connection *loneliness* → *losing confidence* was the third strongest one, while it was the fifth strongest in the second network, and not as strong in the third CLPN network (range: *β* = 0.08 – 0.15). The connection *loneliness* → *thinking of self as worthless* was the fourth strongest in the first network, the third strongest in the second and the fifth strongest in the third CLPN network (range: *β* = 0.10 – 0.14). Moreover, the connection *thinking of self as worthless* → *could not overcome difficulties* was the fifth strongest in the first, the seventh strongest in the second and the third strongest connection in the third network (range: *β* = 0.08 – 0.13). Finally, the connection *losing confidence* → *thinking of self as worthless* was the fourth strongest in the second network, but not as strong in the first and third network (range: *β* = 0.06 – 0.10). The edge weights difference tests for each of the 3 networks indicated significantly stronger connections between the aforementioned edges compared to most other edges (see Figs. S9–S11 in the supplemental material).

**Table 3:** Table depicting the 5 strongest edges for all three networks.

	Network		
edges	pre-COVID to first incidence peak	first incidence peak to second incidence peak	second incidence peak to third incidence peak
1st strongest	thinking of self as worthless → losing confidence	thinking of self as worthless → losing confidence	thinking of self as worthless → losing confidence
2nd strongest	loneliness → feeling unhappy and depressed	loneliness → feeling unhappy and depressed	loneliness → feeling unhappy and depressed
3rd strongest	loneliness → losing confidence	loneliness → thinking of self as worthless	thinking of self as worthless → could not overcome difficulties
4th strongest	loneliness → thinking of self as worthless	losing confidence → thinking of self as worthless	could not overcome difficulties → under stress
5th strongest	thinking of self as worthless → could not overcome difficulties	loneliness → losing confidence	loneliness → thinking of self as worthless

### Symptom centrality

The standardized centrality parameters revealed consistent patterns over time and can be seen in Fig. 2: We found the strongest in-expected-influence for *feeling unhappy and depressed*, followed by *could not overcome difficulties* and *losing confidence*, showing a significantly higher in-expected influence than many other symptoms across sampling times. Lower in-expected-influence values were found for the symptoms *capable of making decisions*, *enjoy normal activities* and *can face up to problems* in all networks and *loneliness* in the CLPN network second incidence peak to third incidence peak (see supplemental material Figs. S12–S14 for in-expected-influence difference tests). The strongest out-expected-influence was found for *loneliness* and *thinking of self as worthless*. The lowest out-expected-influence was found for *feeling reasonably happy*, *can face up to problems*, *playing a useful part* (see supplemental material Figs. S15–S17 for out-expected influence difference tests).

### Results summary

We found consistent patterns of symptom associations over time as *thinking of self as worthless* → *losing confidence* and *loneliness* → *feeling unhappy and depressed* displayed the strongest connections in all networks. Similarly, the standardized centrality parameters indicated that *feeling unhappy and depressed* and *could not overcome difficulties* and *losing confidence* had the strongest in-expected-influence, and *loneliness* and *thinking of self as worthless* had the strongest out-expected-influence in all networks.

## Discussion

Drawing on national, probability-sampled longitudinal data in adults from the UK, this study aimed to disentangle whether mental health symptoms influenced other symptoms or were influenced by other symptoms over time in one network with *maximum psychosocial stress* (pre-COVID to first incidence peak) and in two networks with *repeated, sustained psychosocial stress* (first incidence peak to second incidence peak and second incidence peak to third incidence peak). Loneliness and worthlessness emerged as most central symptoms that lead to the activation of further symptom cascades. As feeling depressed was activated by other symptoms (i.e., high in-expected centrality), this indicates that it is a recipient symptom in the network, constituting a central downstream product of the symptom cascade. Given their influence on other symptoms, low-threshold interventions focusing on self-worth and loneliness may help reduce the spread of network activation.

The present study is the first to analyze a large sample in a longitudinal network design during the COVID-19, which is highly relevant when facing a population stressor like a pandemic. In line with previous studies, psychological distress increased over time from the pre-COVID-19 assessment to the pandemic assessments. Over time, the three resulting networks were consistent as reflected in high correlations between connections among symptoms and the centrality of symptoms. The feeling of worthlessness was among the symptoms with the highest influence on other symptoms (i.e., out-going centrality). In general, self-worth is transdiagnostically associated with mental health [45], but during the pandemic loss of self-worth may be attributable to a variety of reasons such as inability to engage in hobbies or being socially less active [46, 47].

In all three networks loneliness was also among the symptoms with the highest influence on other symptoms in subsequent time-points. Loneliness exerts a strong influence on mental health symptoms of the UK population, which aligns with the findings of Pierce et al. [6] and Kikuchi et al. [11], that during COVID-19 pandemic living alone is associated with poorer mental health, with Groarke et al. [48], that loneliness predicts higher depressive symptoms later in pandemic, and with the cross-sectional network analyses of Wong et al. [19] and Yu and Mahendran [20]. In addition, we extend previous findings by including a longitudinal perspective on individual symptoms.

In line with previous findings before the pandemic that loneliness has an impact on depressive symptoms (for meta-analysis see [49]), the symptoms *feeling unable to overcome difficulties* and *feeling depressed* were affected by loneliness in the present analysis. Intervening on these dynamics appears highly relevant because these patterns could instigate a vicious circle as unhappiness and depressive symptoms are likely associated with less activities and more social withdrawal [50]. Our findings indicate that loneliness before lockdown centrally affects the network during lockdown. This suggests a vulnerability-stress model in that pre-pandemic loneliness leads to the initiation of a symptoms cascade in the presence of a major population stressor. The importance of loneliness and self-worth and their connections among each other (e.g., *loneliness* → *thinking of self as worthless*) align with the sociometer theory of self-esteem that self-esteem functions as an internal proxy of social inclusion [51]. Thus, these symptoms can be understood as the cluster sense of belonging. Maintaining and restoring this basic human need of belonging during lockdowns is thus of paramount importance.

Surprisingly, enjoying day to day activities did not emerge as key symptom despite substantial restrictions to engage in hobbies or social activities. This indicates that the cognitive evaluation and general coping mechanisms may be decisive for initiating symptom dynamics and not the restrictions per se [52]. In line with this, cognitive reappraisal interventions have been found to strengthen resilience against deterioration in positive mood caused by being alone [53].

### Clinical implications

The connections and the central symptoms with their connections can be used to investigate, both through experimental and clinical studies, whether these are indeed causal for symptom escalation under stress. Subsequently, it is to be examined whether this symptom escalation, i.e., this symptom spread can be prevented by addressing these nodes [14, 54, 55].

Together with previous findings on loneliness [48, 56, 57], our study reemphasizes the importance of loneliness for the escalation of other mental health symptoms, thus constituting a key target for intervention [58]. Prevention and intervention programs could be implemented on societal level (e.g., in educational institutions) as regular courses on stress reduction, emotional regulation, empathy, and self-compassion after proving their effectiveness. On an individual level, home visits and daily contact programs were proposed to target loneliness [58]. Hence, at any time in the pandemic, especially at the relevant times of onset and incidence peaks, it is advisable and important to address the social connectedness and potential social isolation of individuals and to implement appropriate programs to counteract the impairment of mental health at an early stage. For instance, when implementing contact restrictions, policy makers need to consider how to adequately enable social support groups or small in-person group activities to stop the manifestation of loneliness. For example, single-session growth mindset interventions for adolescent anxiety and depression could be helpful to reduce mental health deterioration [59].

The results of our study revealed that, prior to the COVID-19 pandemic, GHQ-12 mean levels were slightly above the clinical cut-off of 11 as established by Goldberg et al. [31]. In the following three waves during the pandemic, the threshold was likewise exceeded with an increase of one point from the pre-COVID-19 survey (see [60] for a more detailed analysis on clinical change in this sample). Despite the small effect sizes of the observed changes, it is important to note that this is a population-based study and small effects can have a significant impact on a large population [61]. The present study provides insights into the dynamics of symptom levels, and sheds light on the activation of symptom cascades that may have been involved in such population level shifts. It appears that the symptom cascades and the findings as a whole have a high relevance in our sample, and we found that this relevance is not only for the subjects who were already in the pathological range (measured with the GHQ-12), but also for the subjects with subclinical mental health issues.

In addition, it is important for prevention and intervention to disentangle how to address the needs for individuals who were already lonely before the pandemic compared to persons who became lonely in light of the restrictions [56]. From our findings it can be deduced that loneliness does not have to be addressed only during the pandemic, but already before, so that one is better prepared for such stressors [58]. As loneliness was an important variable during all stages of the pandemic, it needs to be investigated whether different groups of individuals felt lonely during the pandemic. As several studies found younger individuals to be at a higher risk (e.g. [57, 62]), prevention and intervention programs should address these groups in particular. Li & Wang [63] analyzed the first wave of the Understanding Society dataset with weighted univariate analyses. They found significantly higher scores in GHQ-12 and for loneliness among women, younger people, participants without a partner, and unemployed individuals. These findings suggest that these subgroups represent populations at higher risk, warranting the implementation of specific intervention and preventive measures tailored to these populations. Intervention programs that take place online are already available for this purpose (e.g. [64, 65]), reducing loneliness and ameliorating feelings of belonging (for an extended review see ref. [66]). Single-session interventions are also available, and first studies found evidence of reducing hopelessness and increasing agency, among other outcomes [67]. There are also targeted programs aimed at reducing loneliness, such as the digital intervention “Happify”, which has been shown to have a significant positive impact on feelings of loneliness [68]. Also, group interventions conducted via Zoom have shown positive significant effects on loneliness and depression [69]. Similarly, interventions can be implemented that can reduce worthlessness and enhance self-worth [70]. Overall, there are different opportunities to implement appropriate specific interventions to counteract the deterioration of symptom cascades at an early stage. These could be implemented as early interventions or, at the onset of a pandemic, in terms of indicated prevention as a preventive measure for potential at-risk groups.

Targeting specific reasons of loss of self-worth may be advisable for intervention efforts, as loss of self-worth may be attributable to a variety of reasons such as inability to engage in hobbies or being socially less active [46, 47]. The question of appropriate interventions here is a particularly individual one that depends on the cause of the loss of self-worth. In the case of the loss of employment, support for existential issues would be more likely helpful. The inability to engage in hobbies might be more likely to benefit from motivation to resume previous hobbies or to take up new ones. Reduced social activity with its negative impact on self-worth can be addressed closely in conjunction with the interventions mentioned above for loneliness.

### Strengths and limitations

A major advantage of our study is the use of a representative, large dataset that includes both pre-pandemic surveys and waves during the pandemic. It is particularly advantageous that these waves took place during pandemic-relevant assessment intervals, so that, based on the stringency index, particularly relevant time points could be examined.

The network analytic approach allowed us to perform an analysis with a high degree of resolution, as we can describe dynamic changes over time and differentiate these changes in incoming and outgoing connections separately (influence of vs. on a symptom). This is important, as the common cause perspective of mean value differences of GHQ-12 suggests targeting psychological distress holistically, which can be complicated, while we provide specific target points by identifying constitutive elements of psychological distress.

One limitation results from the necessary use of imputation. Missingness was a concern in the present study. Our conclusions hold only under the missing at random (MAR) assumptions, which is based on our own missingness analyses and previous studies on this dataset that identified important variables associated with missingness (e.g. [6]). Although our results point to plausible intervention targets, within- and between person variance remains entangled in the present study [37], and intervention studies are needed first to showcase the effects of targeting central symptoms (e.g. refs. [14, 54]). The results obtained here relate specifically to pandemic events and conditions in the UK. Because the pandemic event was a country-specific and individual event for which country-specific measures were taken, our results cannot be reliably extrapolated to other countries or populations. As the GHQ-12 is primarily tailored to depressive symptoms, it would be useful to broaden the range of symptoms in further studies by including anxiety symptoms or more fine-grained loneliness measures to unravel specific dynamics among these symptoms. In the Understanding Society study, loneliness was assessed with a single item, limiting the construct validity of loneliness. While this item presents an indicator of the subjective feeling of loneliness, important nuances underlying loneliness and their role in an evolving network remains masked. We cannot discern differential effects of important aspects of social loneliness like a lack of social contact and emotional loneliness or a lack of emotional closeness and intimacy within a network perspective. These other facets of loneliness should be considered in future studies, for instance by the means of the UCLA 3-item loneliness scale as an approximate measure or other more extensive scales [71, 72].

Furthermore, it is important to note that the GHQ-12 is a screening tool to indicate caseness but does not possess psychiatric diagnostic-specific validity. Nevertheless, the GHQ-12 is useful for capturing important transdiagnostic symptoms commonly found across several mental disorders. This allowed us to examine important associations between these transdiagnostic symptoms and loneliness. However, the change in survey method from face-to-face surveys to online surveys or telephone surveys must be critically considered [73]. Dropout in 2020 was particularly higher in the older age groups, among people living alone and among people with a lower level of education. In addition to the reduced response rate, it cannot be ruled out that changes in the survey format induced response bias. However, a thorough psychometric analysis of the GHQ-12 in the present sample indicated longitudinal measurement invariance across time before and during COVID-19 [73].

## Conclusion

Our study provides important insights concerning the influence of symptoms on future symptoms and symptom constellations of psychological distress. The present findings suggest that feelings of loneliness and worthlessness increase the risk of mental health deterioration and facilitate later cascades of symptom escalation under chronic stressors such as repeated lockdowns, thus constituting clinically relevant targets for prevention and intervention. Feeling depressed and not overcoming difficulties had many incoming connections, thus constituting an end-product of symptom cascades. Further research is needed to investigate whether and how our findings can be translated into clinical practice and how they may inform policy makers. The associations we found can be used to investigate, through experimental and through clinical studies, whether these nodes are causal for symptom escalation under stress and whether this symptom escalation can be prevented by addressing these nodes.

## Supplementary information

Supplemental Material
