# Supplementary material for: Temporal dynamics in mental health symptoms and loneliness during the COVID-19 pandemic in a longitudinal probability sample: a network analysis
Source: Transl Psychiatry. 2023 May 10;13:162. doi: 10.1038/s41398-023-02444-z (PMC10170425; doi:10.1038/s41398-023-02444-z)

| **Pre-COVID to first incidence peak**  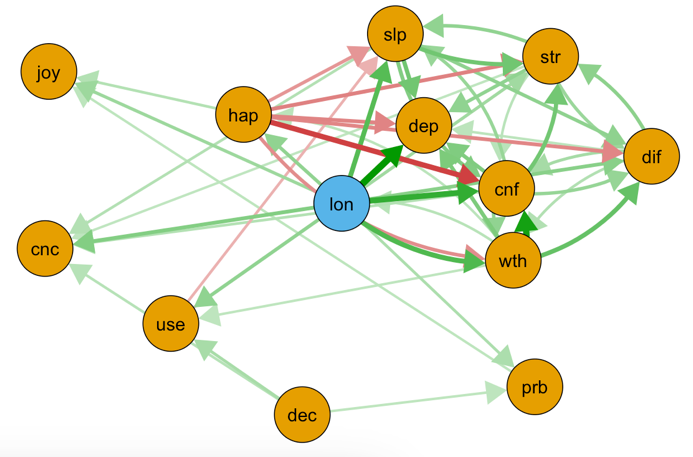 | **First incidence peak to second incidence peak**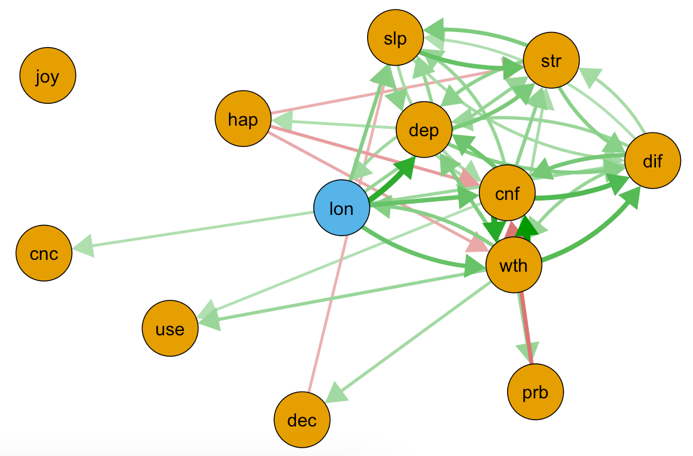 |
| --- | --- |
| **Second incidence peak to third incidence peak**  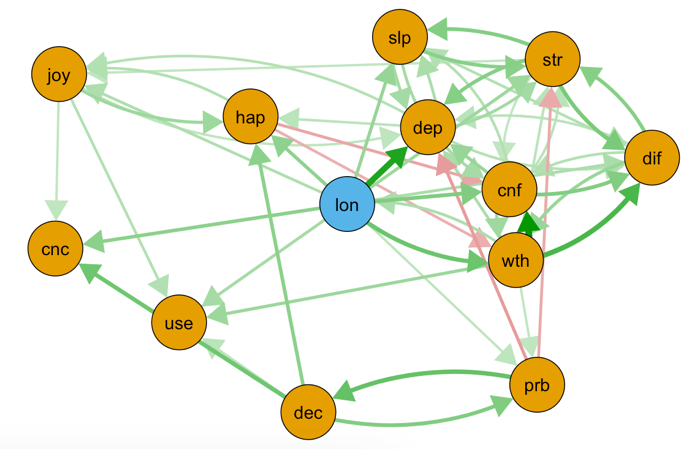 | - cnc = able to concentrate - cnf = losing confidence - dec = capable of making decisions - dep = feeling unhappy and depressed - dif = could not overcome difficulties - hap = feeling reasonably happy - joy = enjoy normal activities - prb = can face up to problems - slp = lost much sleep - str = under stress - use = playing a useful part - wth = thinking of self as worthless - lon = feeling lonely |

**Figure s1.** The cross-lagged panel networks with complete cases without imputed data for pre to incidence peak and incidence peak to incidence peak time-points. The relationship of the symptoms is indicated by the arrow’s color (green = positive, red = negative), the strength of the relationship is indicated by the arrow’s thickness (thicker = stronger). In these networks, autoregressive effects are excluded. Threshold was set to .05, excluding all relationships < .05. Final *N*= 7,815


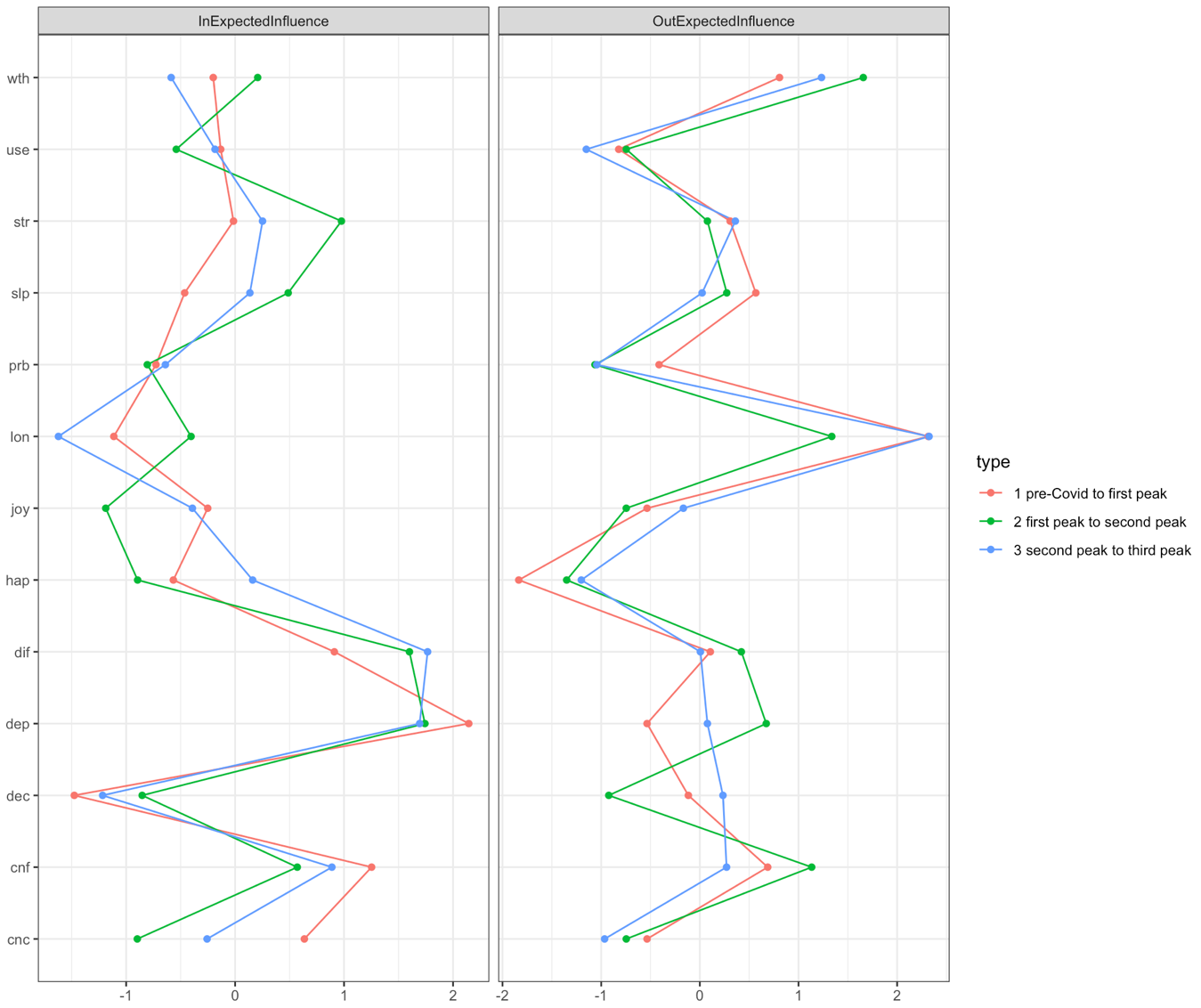


thinking of self as worthless

playing a useful part

under stress

lost much sleep

can face up to problems

feeling lonely

enjoy normal activities

feeling reasonably happy

could not overcome difficulties

feeling unhappy and depressed

capable of making decisions

losing confidence

able to concentrate

**Figure s2.** Symptom centrality estimates for the networks with complete cases without imputed data using z-values. Greater values indicate greater centrality. Out-expected-influence is the degree to which a symptom predicts other symptoms at the subsequent relevant point. In-expected-influence is the degree to which a symptom is predicted by other symptoms at the subsequent relevant point. Final *N*= 7,815


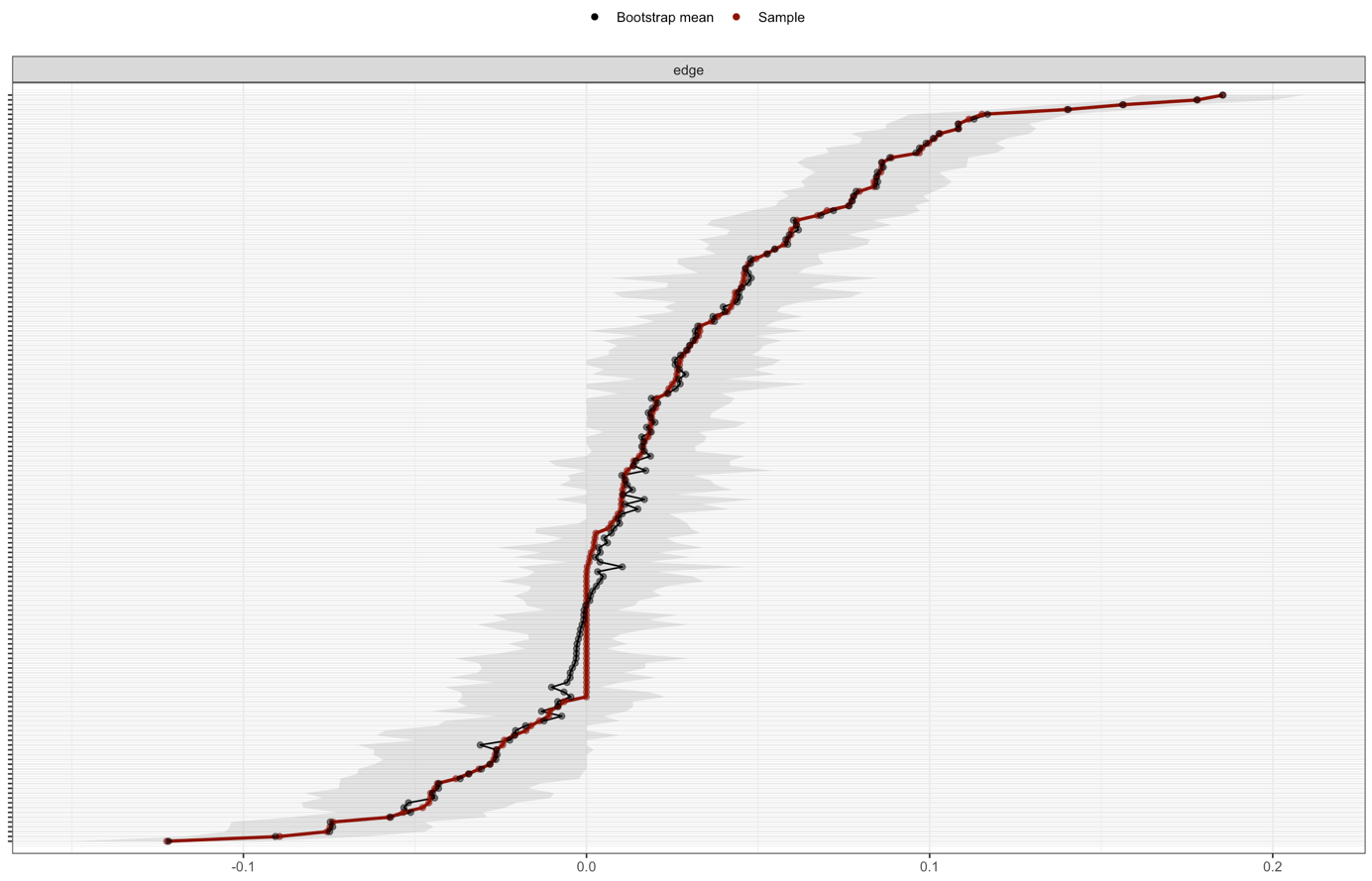


**Figure s3.** GHQ-12 and loneliness item interrelation accuracy plots for the Pre-Covid (P1) → First Covid incidence peak (T1) network with 1000 bootstrap iterations. The plot shows the sample interrelations (i.e., edge weights; red dots), the means of the bootstrapped interrelations (i.e., edge weights; black dots), and the bootstrap confidence intervals.

**
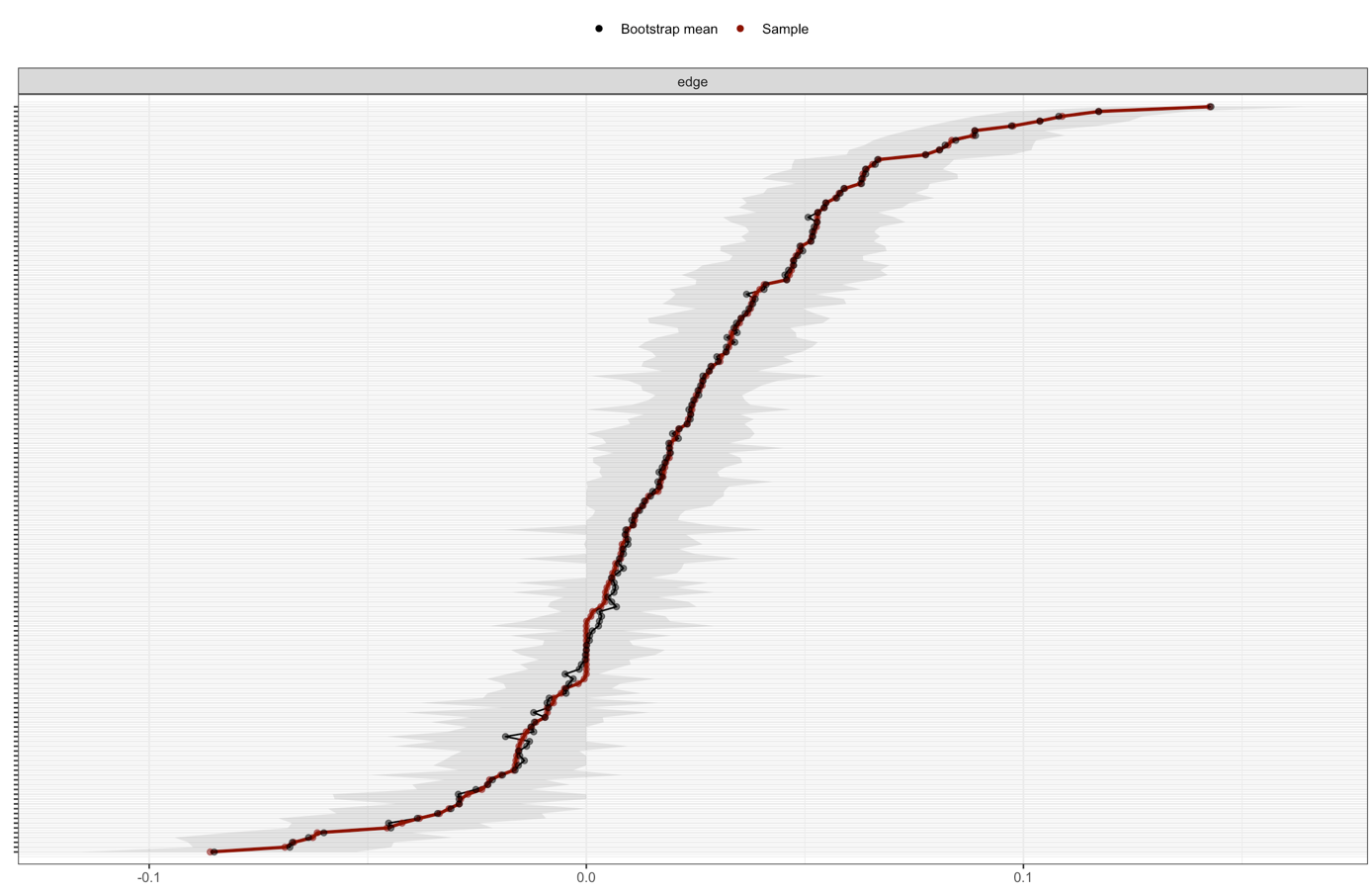
**

**Figure s4.** GHQ-12 and loneliness item interrelation accuracy plots for the T1 → Second Covid incidence peak (T2) network with 1000 bootstrap iterations. The plot shows the sample interrelations (i.e., edge weights; red dots), the means of the bootstrapped interrelations (i.e., edge weights; black dots), and the bootstrap confidence intervals.


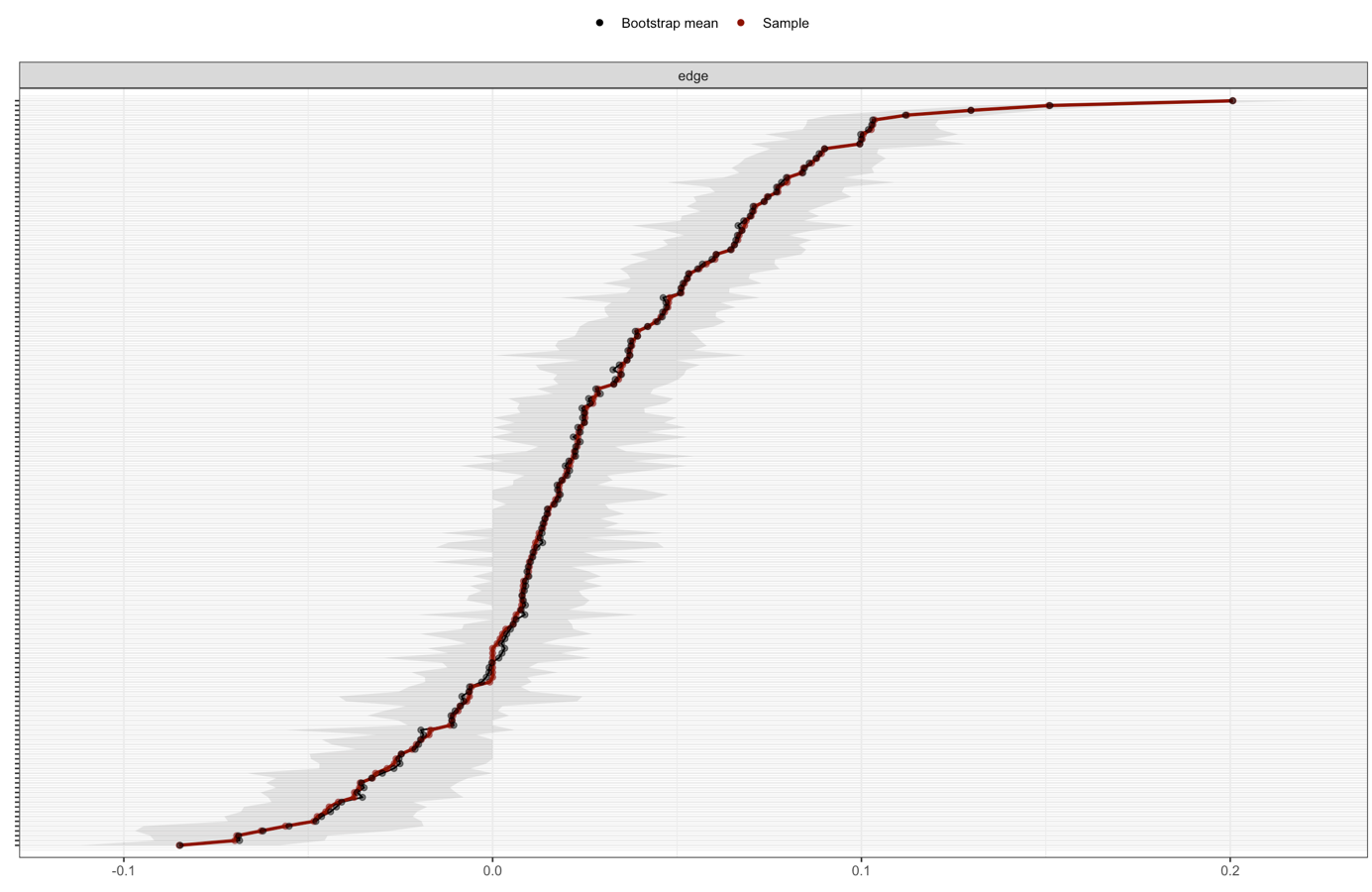


**Figure s5.** GHQ-12 and loneliness item interrelation accuracy plots for the T2 → Third Covid incidence peak (T3) network with 1000 bootstrap iterations. The plot shows the sample interrelations (i.e., edge weights; red dots), the means of the bootstrapped interrelations (i.e., edge weights; black dots), and the bootstrap confidence intervals.

**
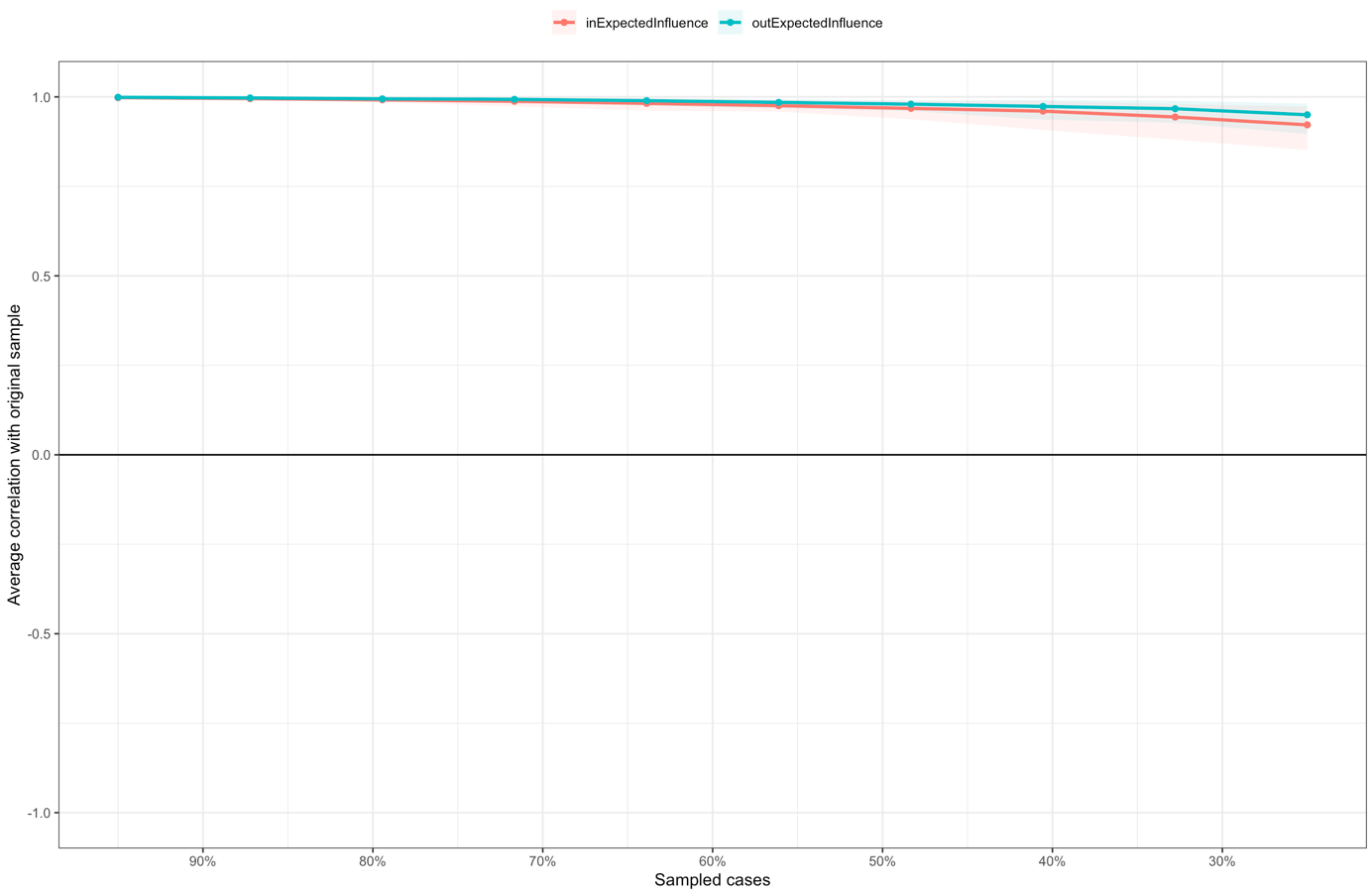
**

**Figure s6.** Stability of centrality measures for the P1 → T1 network


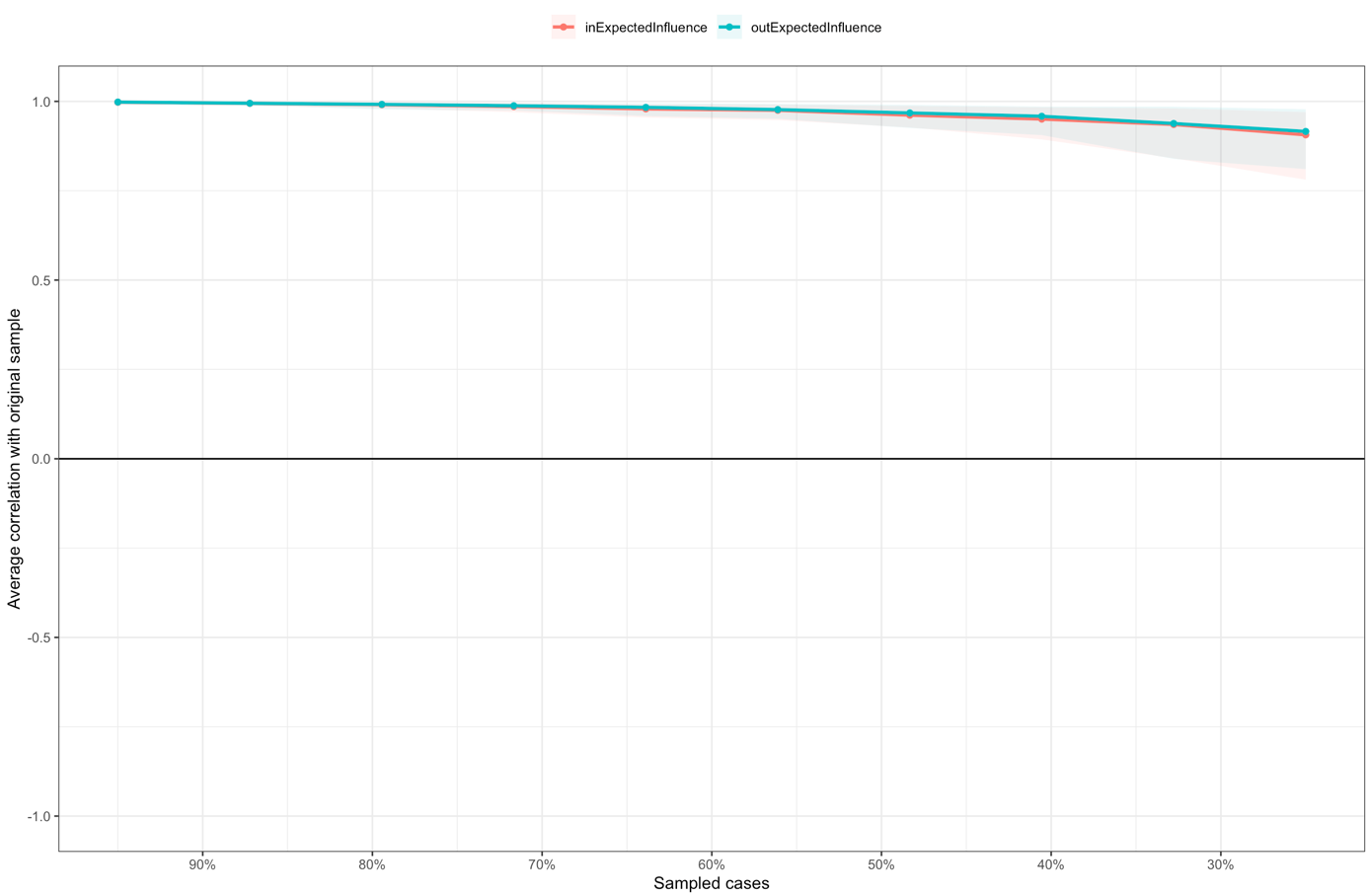


**Figure s7.** Stability of centrality measures for the T1 → T2 network


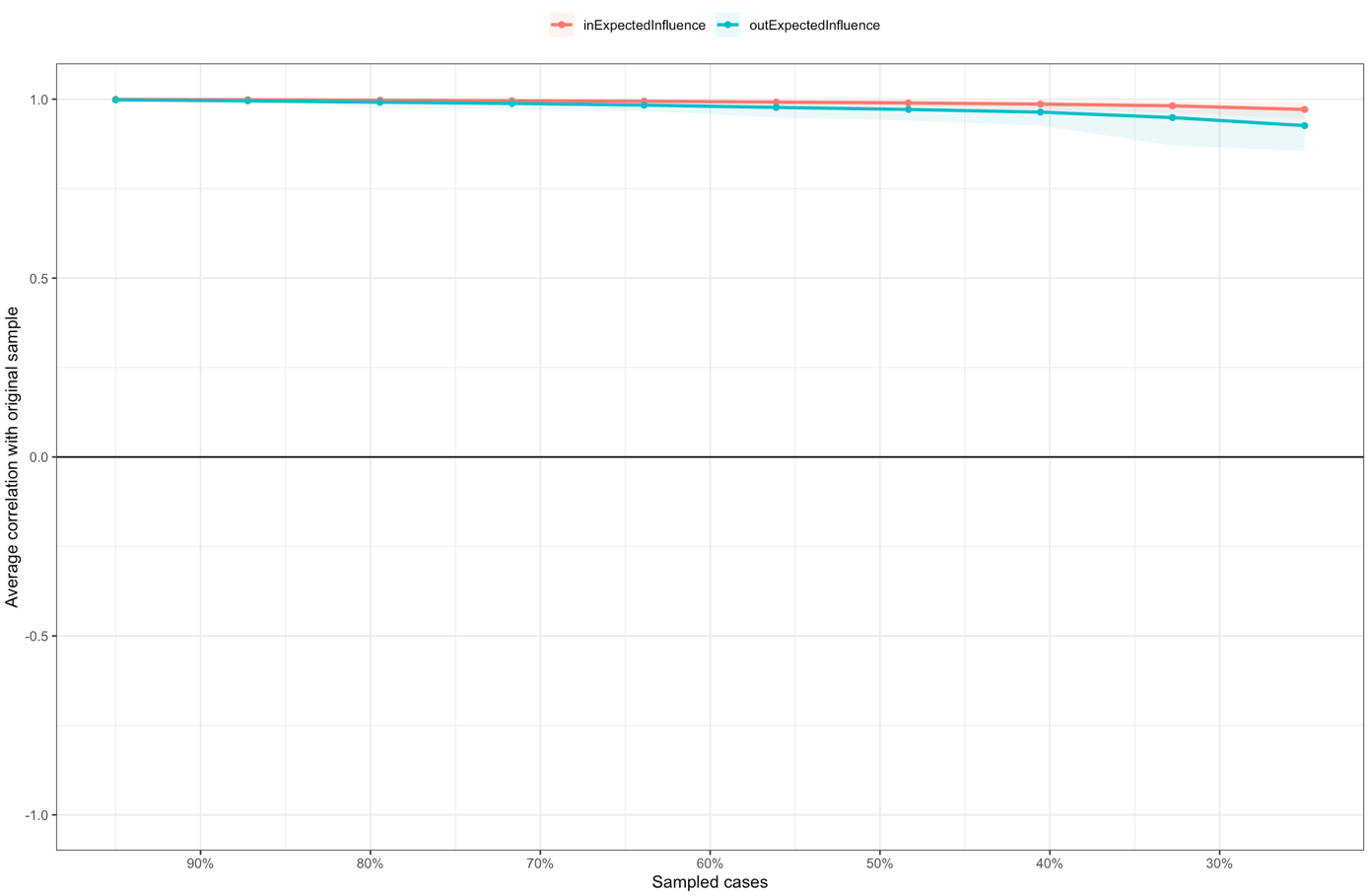


**Figure s8.** Stability of centrality measures for the T2 → T3 network

**
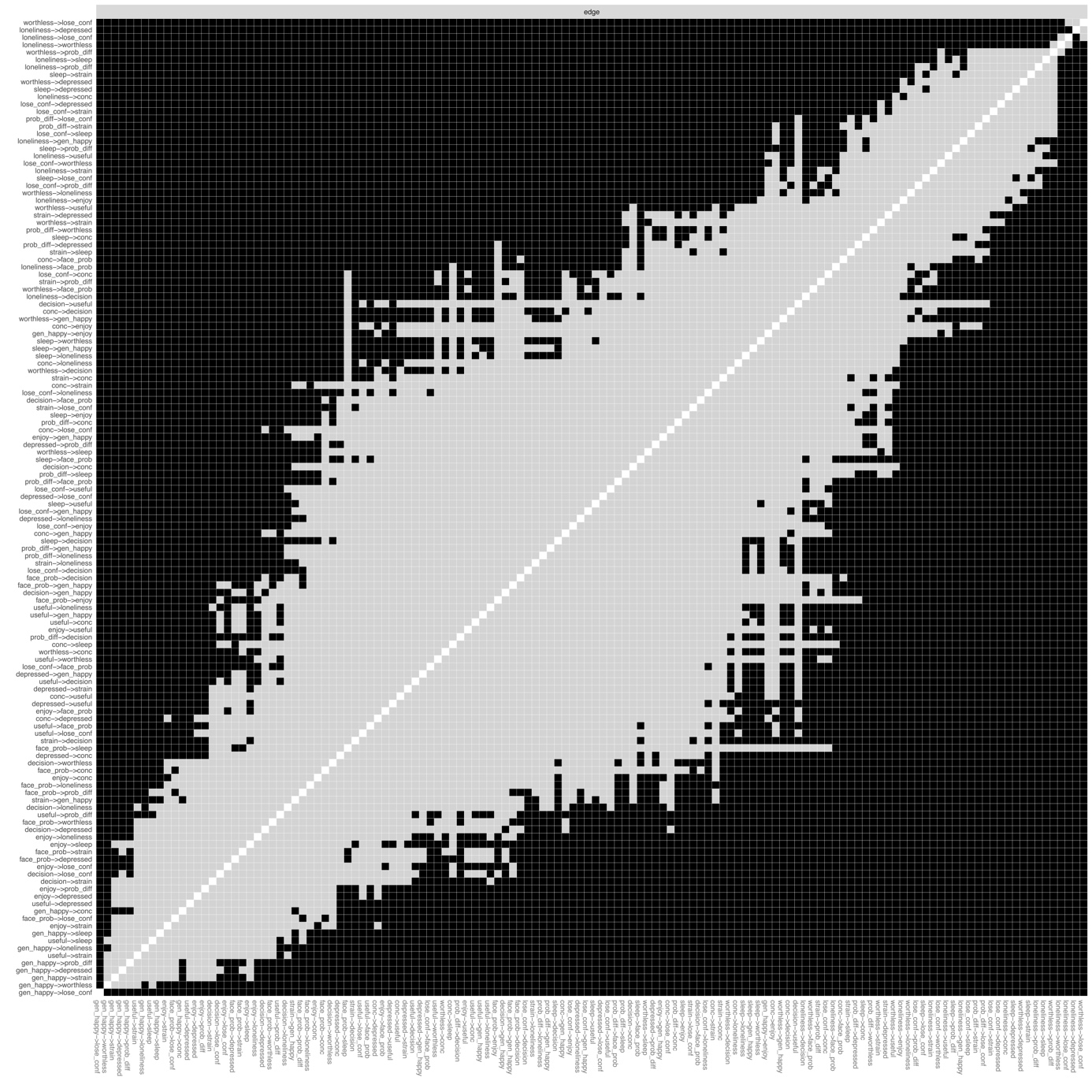
**

**Figure s9.** Edge weight difference tests for the P1 → T1 network with black boxes indicating edges that significantly differ from each other (*p* < .05) and gray boxes indicating no differences.

**
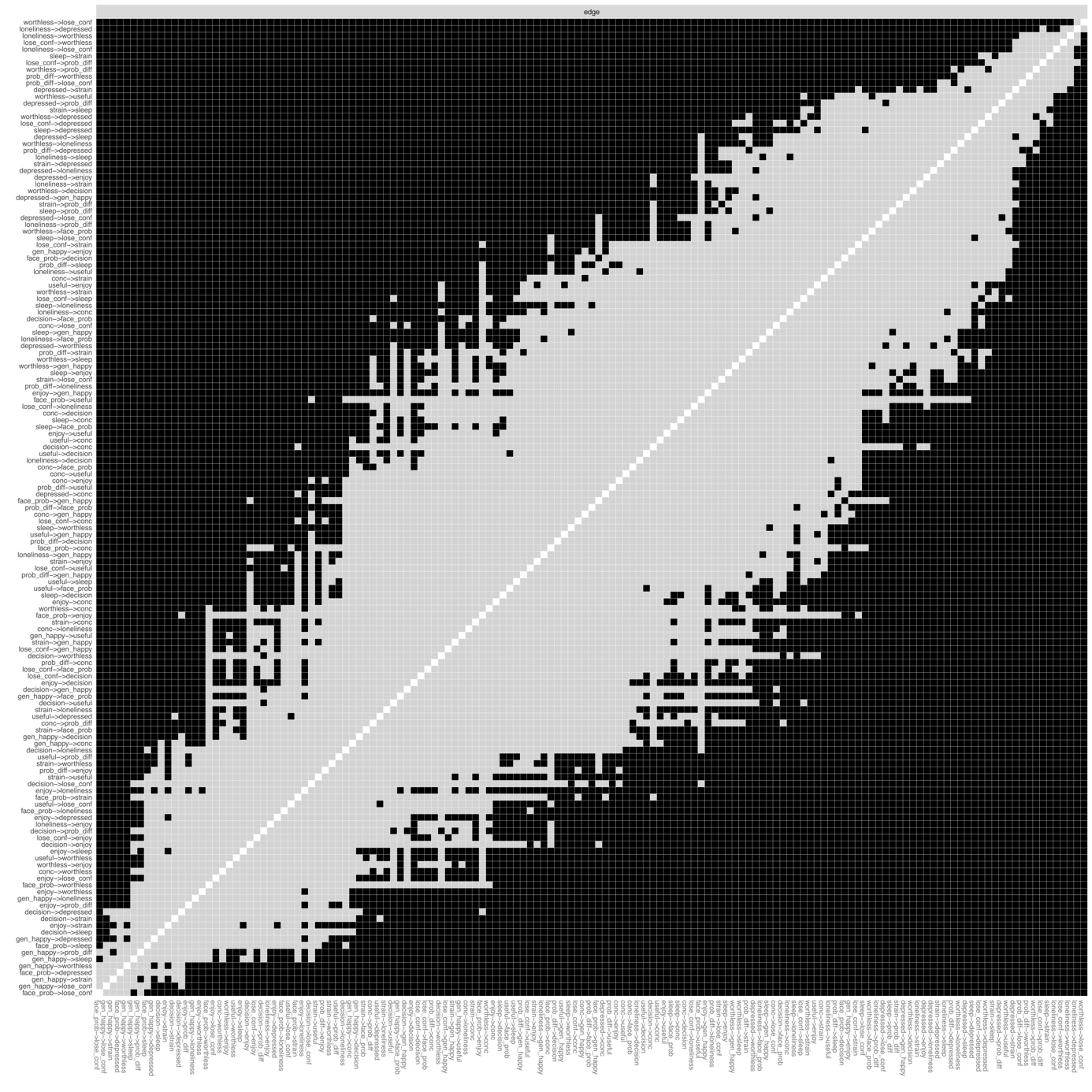
**

**Figure s10.** Edge weight difference tests for the T1 → T2 network with black boxes indicating edges that significantly differ from each other (*p* < .05) and gray boxes indicating no differences.

**
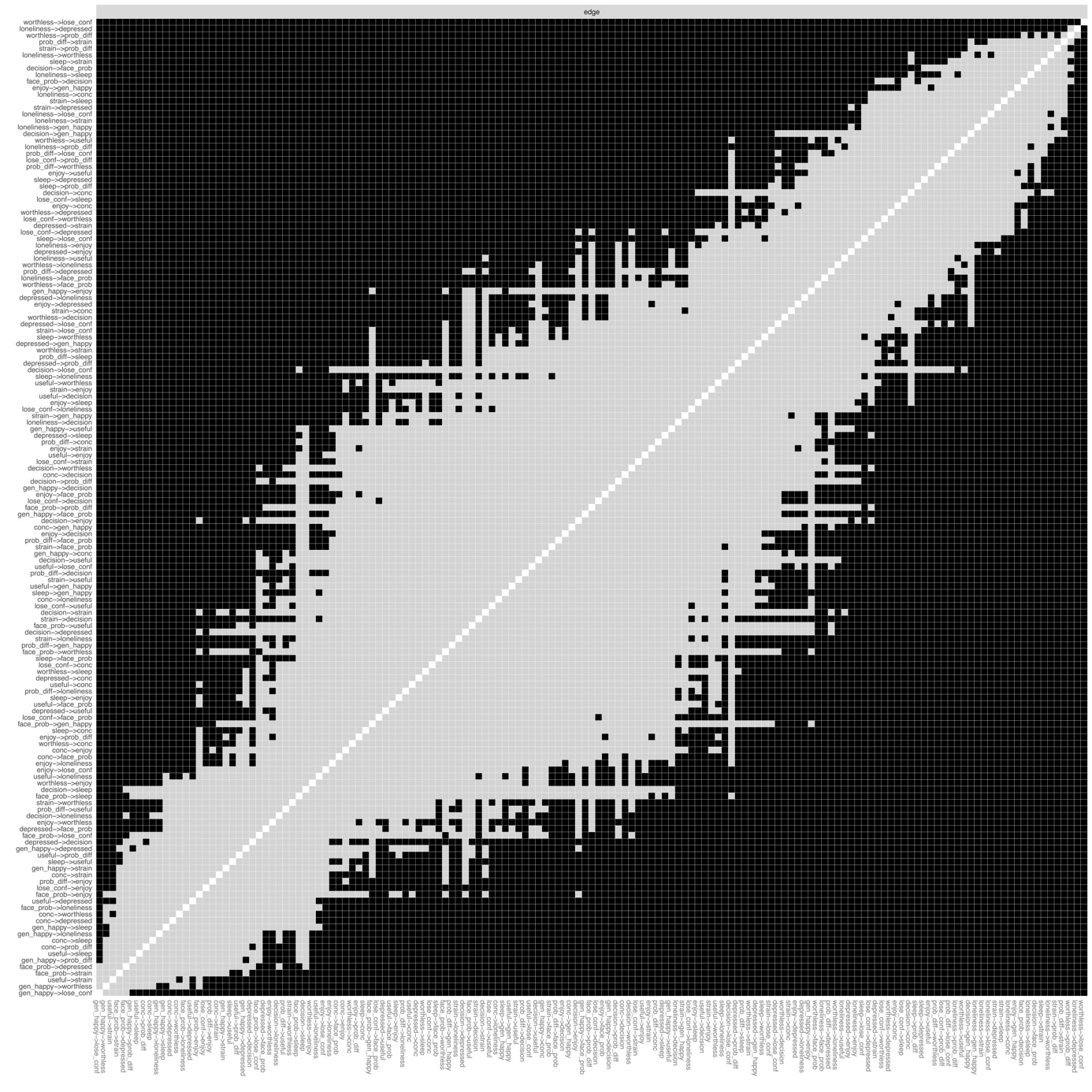
**

**Figure s11.** Edge weight difference tests for the T2 → T3 network with black boxes indicating edges that significantly differ from each other (*p* < .05) and gray boxes indicating no differences.


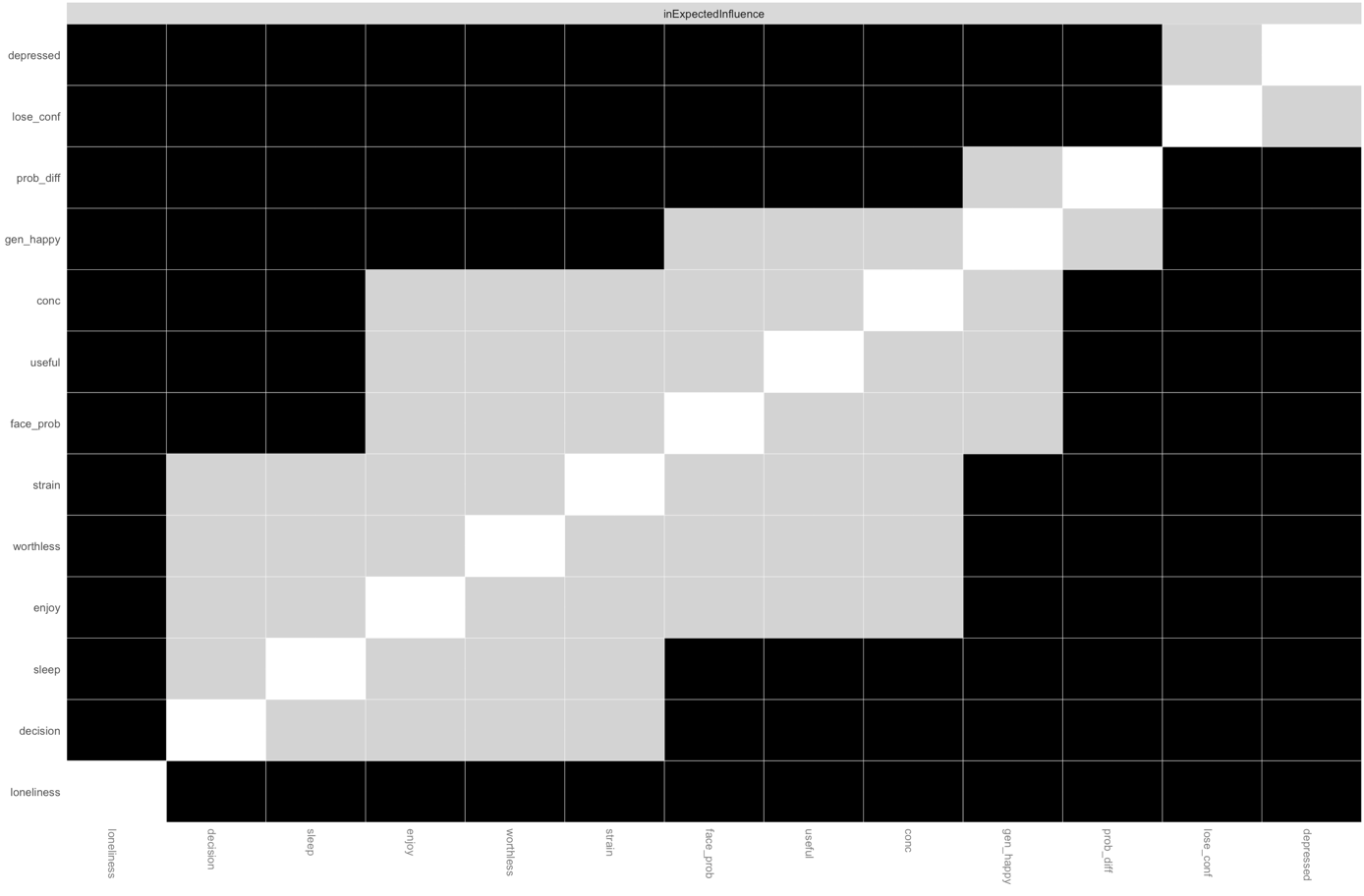


**Figure s12.** In-expected-influence difference tests for the P1 → T1 network with black boxes indicating edges that significantly differ from each other (*p* < .05) and gray boxes indicating no differences.


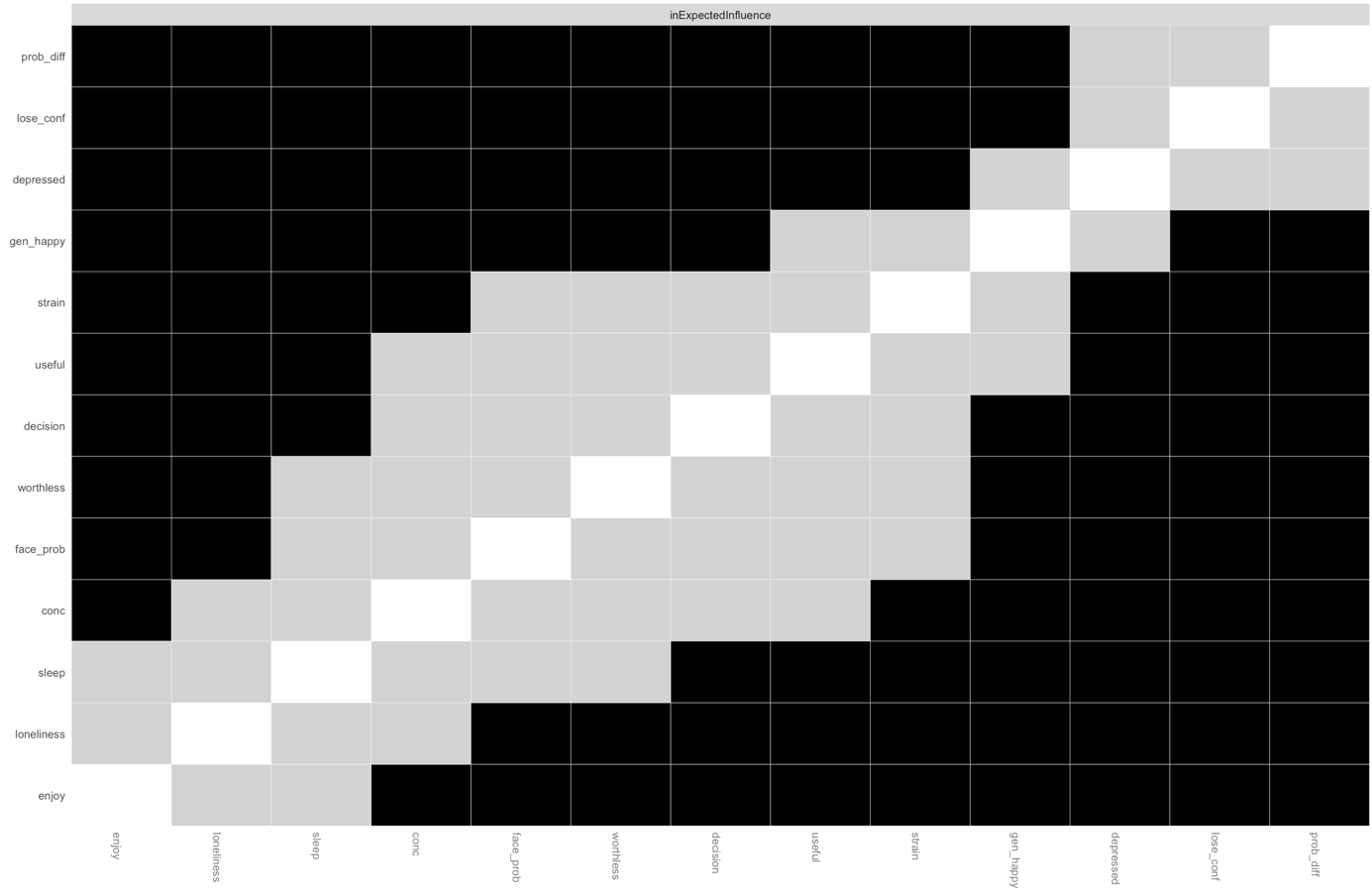


**Figure s13.** In-expected-influence difference tests for the T1 → T2 network with black boxes indicating edges that significantly differ from each other (*p* < .05) and gray boxes indicating no differences.


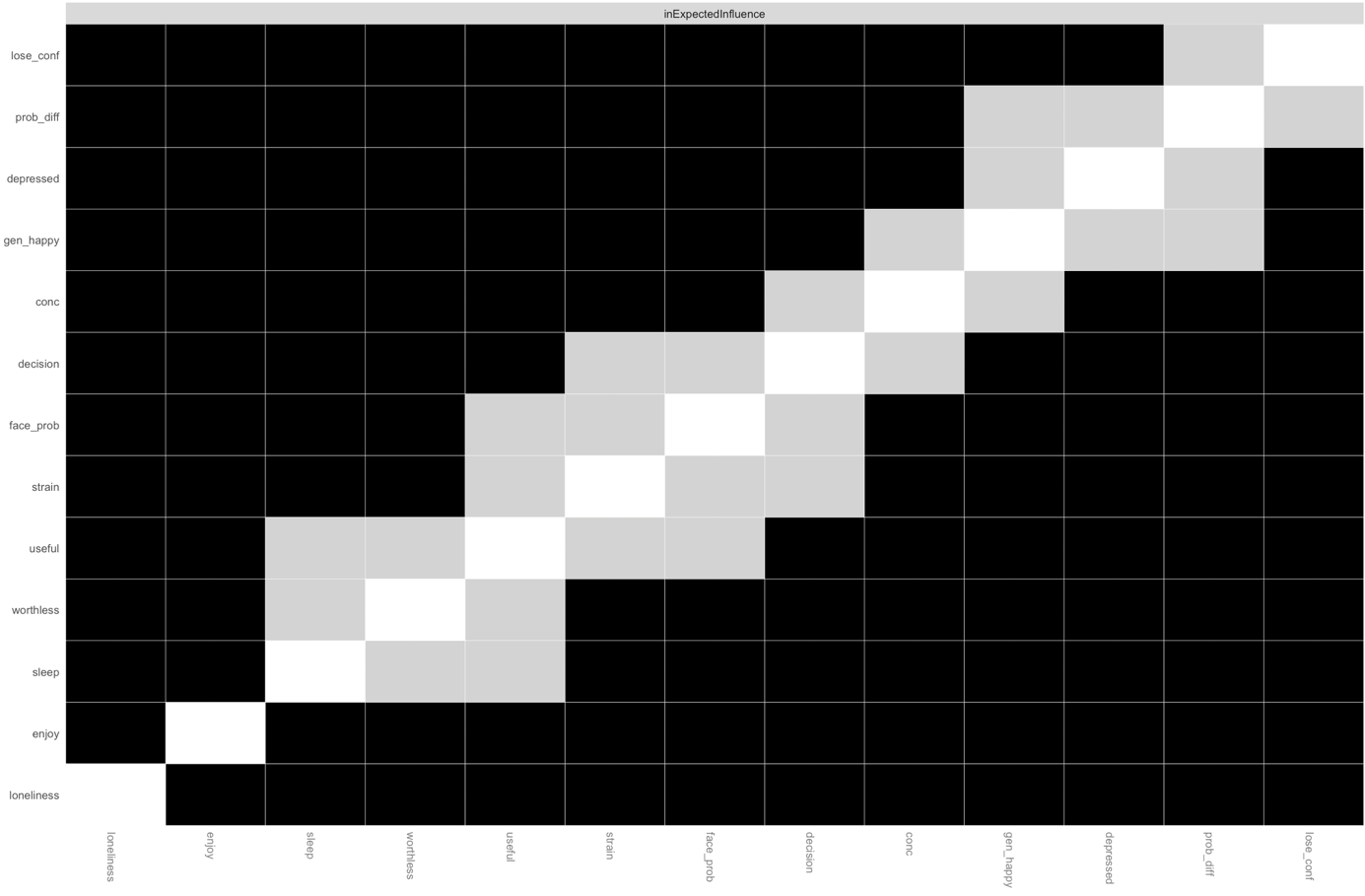


**Figure s14.** In-expected-influence difference tests for the T2 → T3 network with black boxes indicating edges that significantly differ from each other (*p* < .05) and gray boxes indicating no differences.


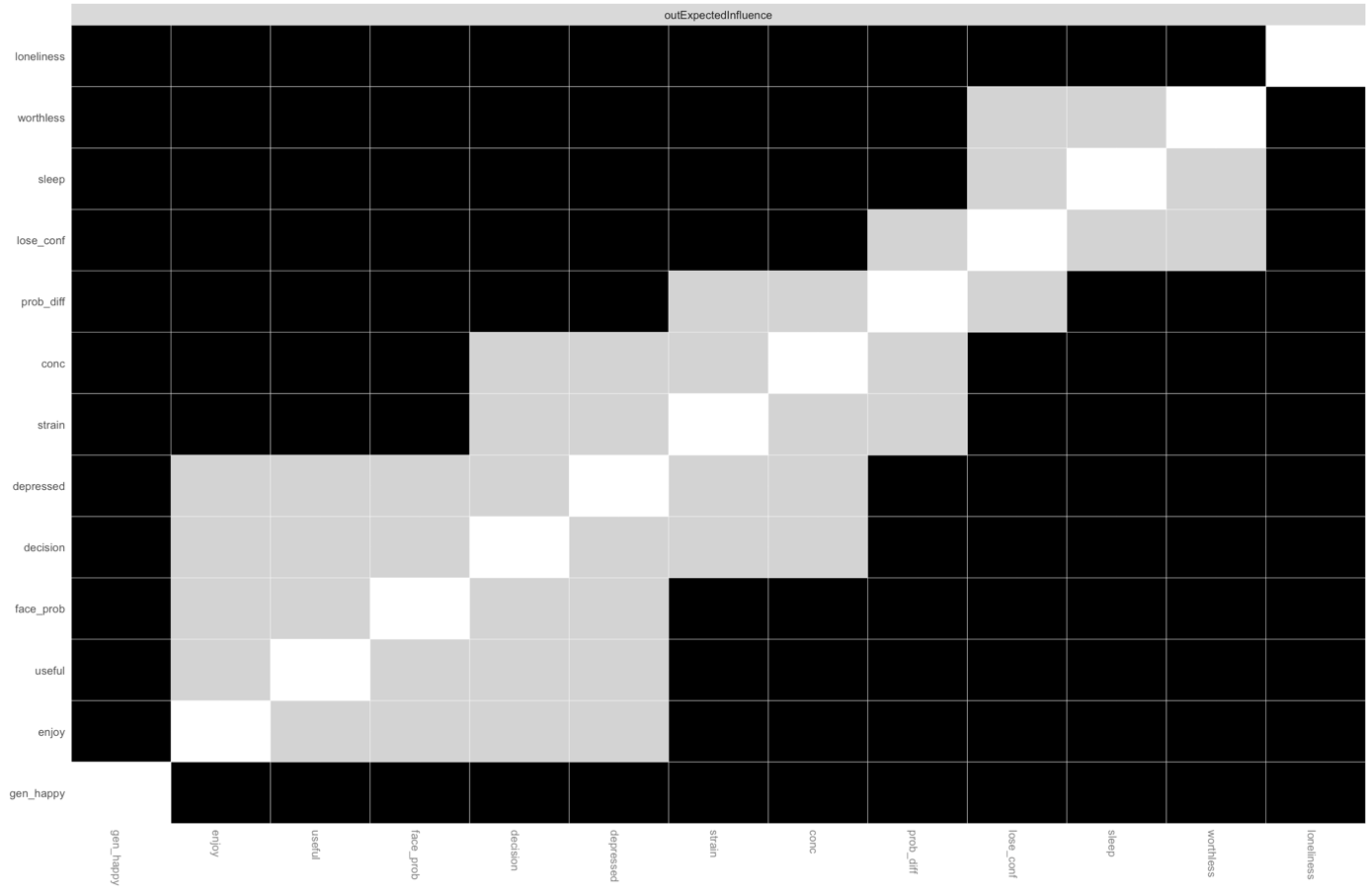


**Figure s15.** Out-expected-influence difference tests for the P1 → T1 network with black boxes indicating edges that significantly differ from each other (*p* < .05) and gray boxes indicating no differences.


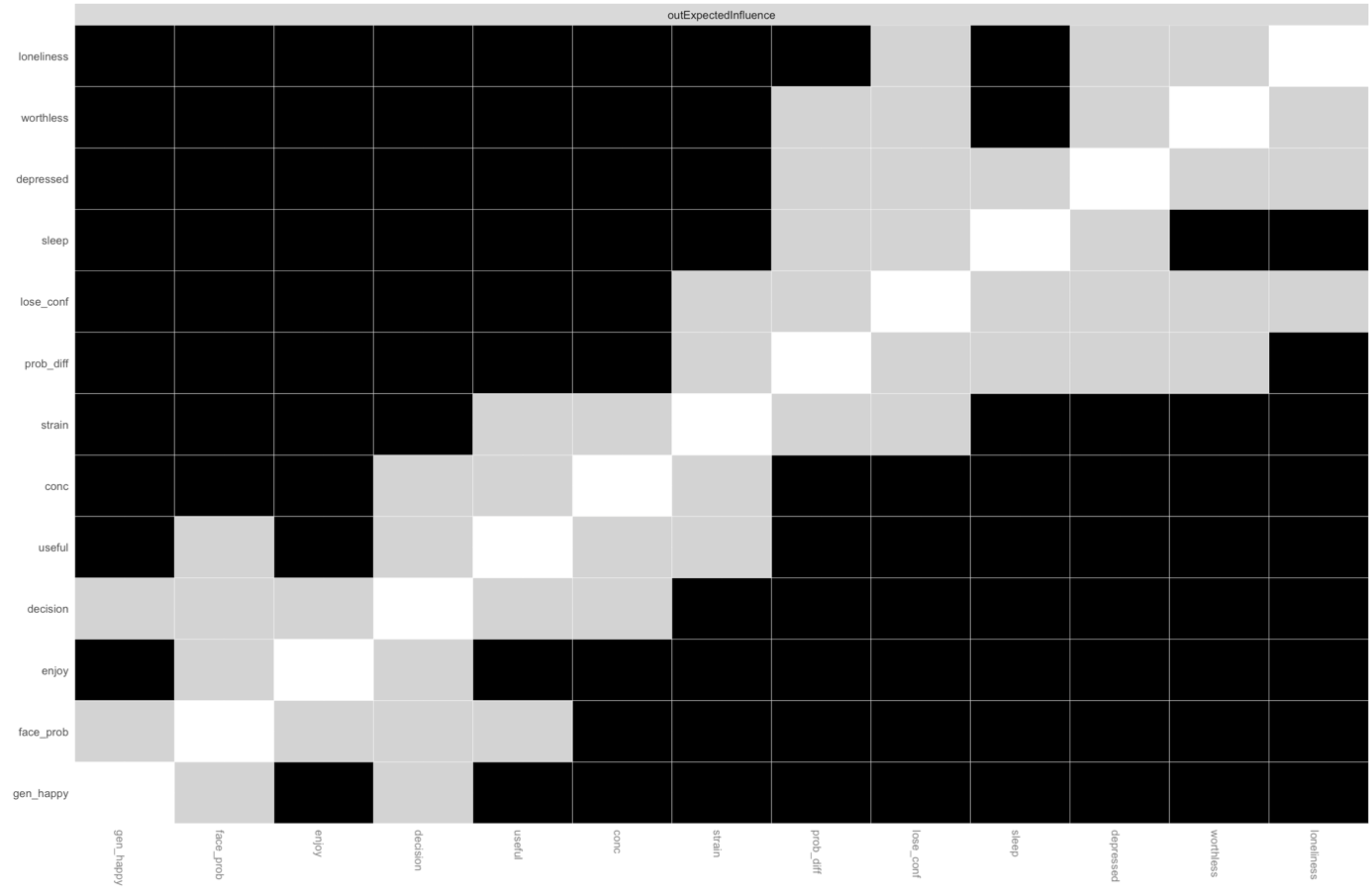


**Figure s16.** Out-expected-influence difference tests for the T1 → T2 network with black boxes indicating edges that significantly differ from each other (*p* < .05) and gray boxes indicating no differences.


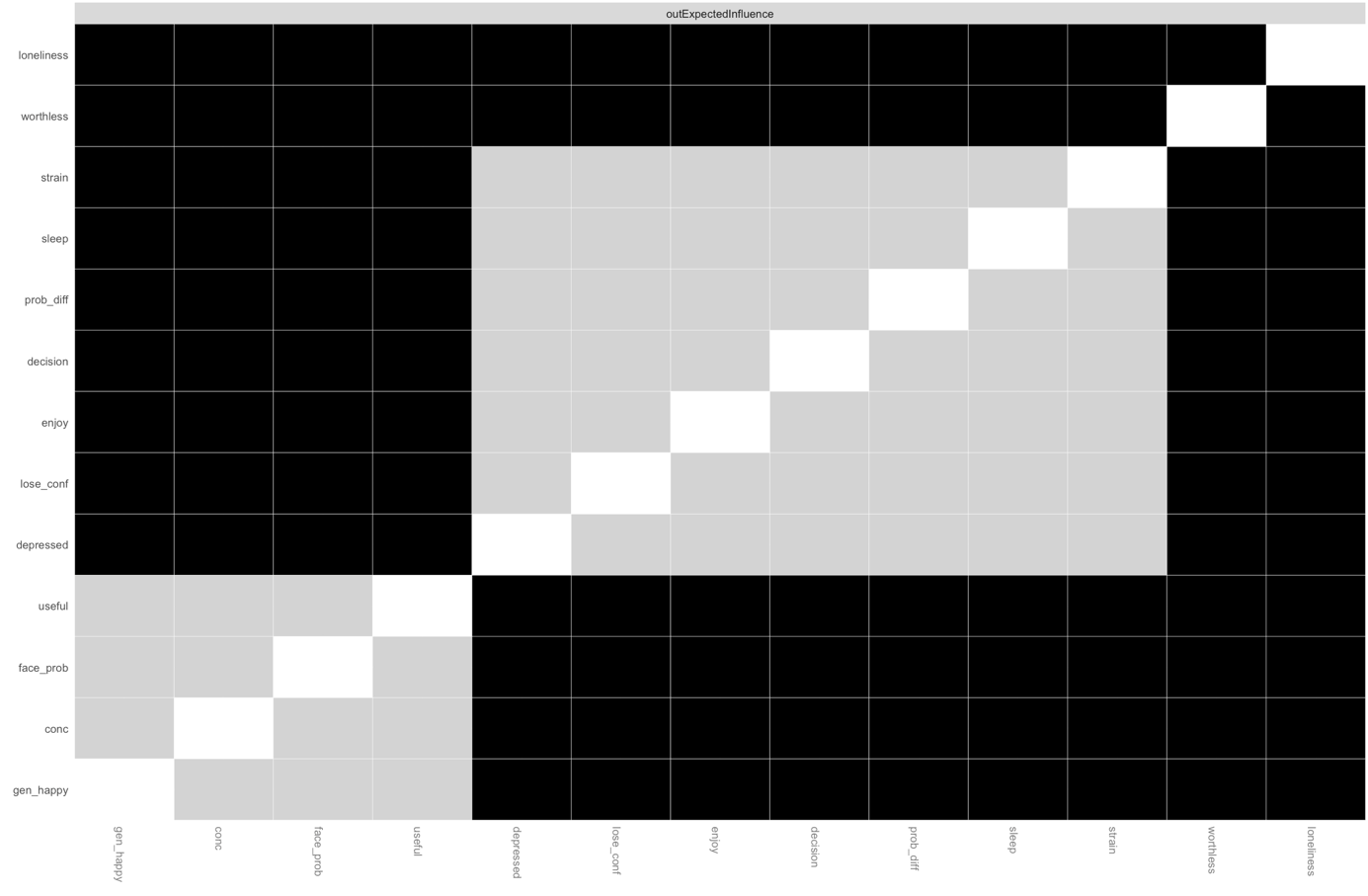


**Figure s17.** Out-expected-influence difference tests for the T2 → T3 network with black boxes indicating edges that significantly differ from each other (*p* < .05) and gray boxes indicating no differences.


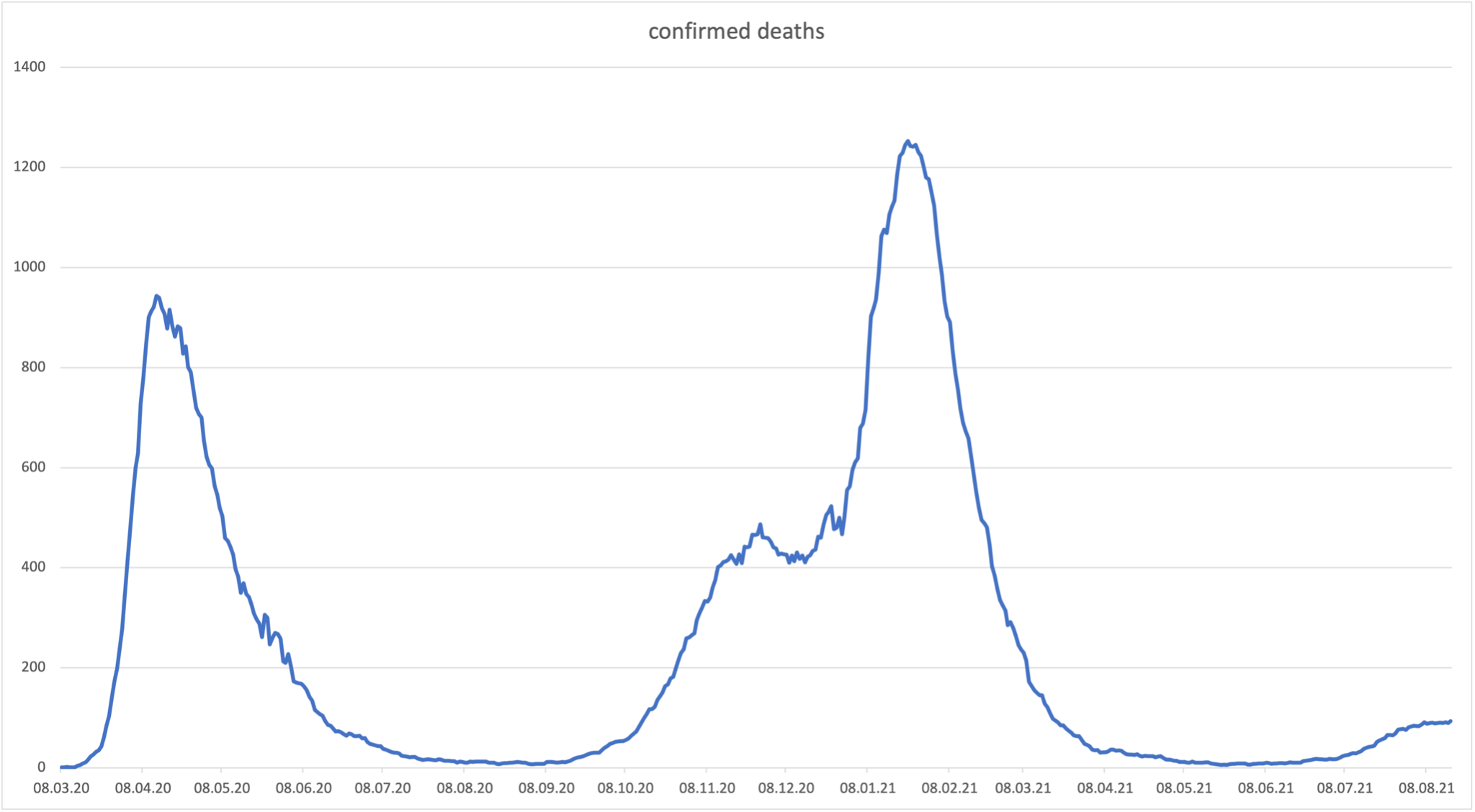


**Figure s18.** Confirmed COVID-19 deaths in the UK beginning in March 2020 until August 2021. Data from https://ourworldindata.org/coronavirus#explore-the-global-situation


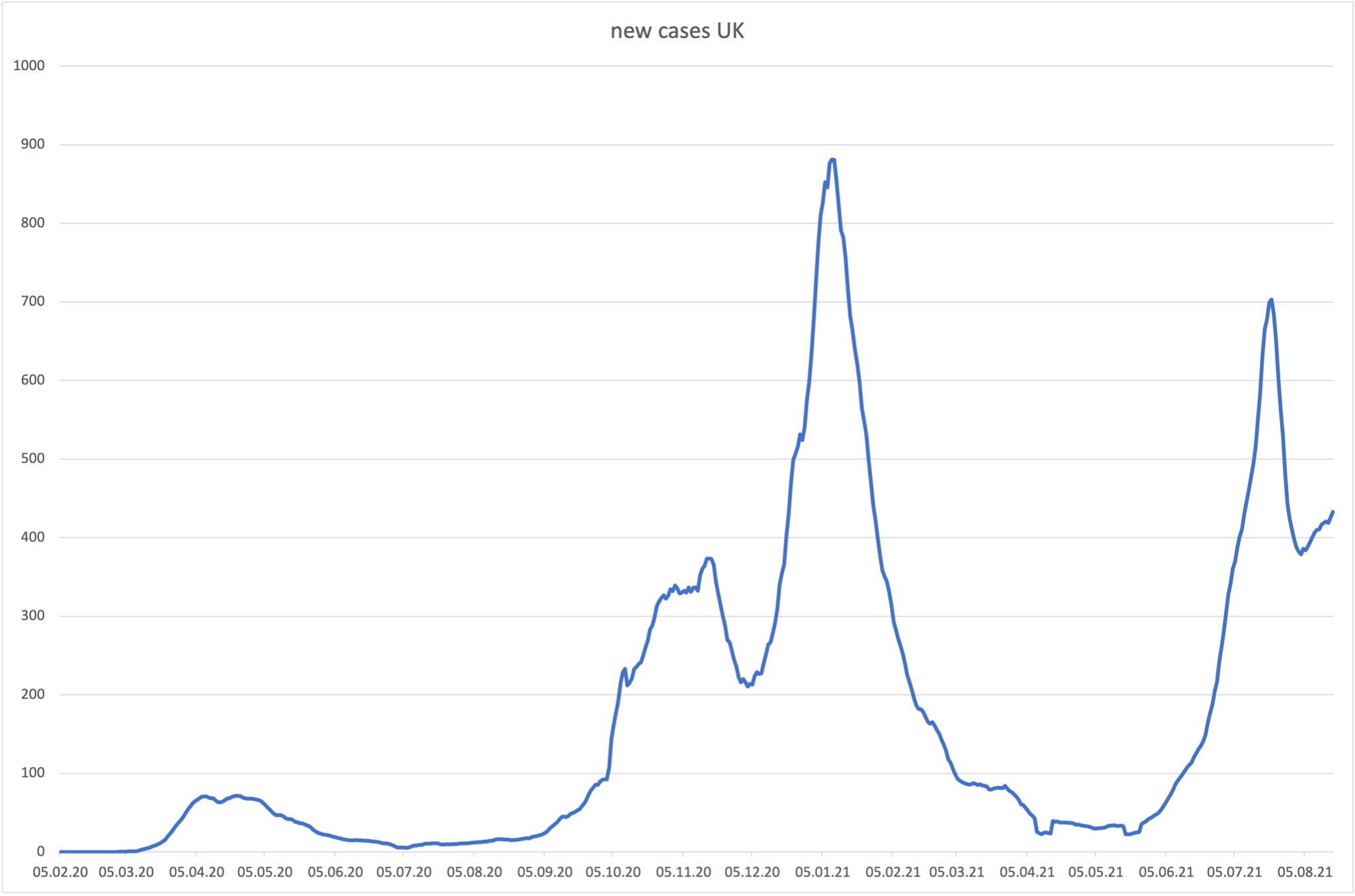


**Figure s19.** Confirmed COVID-19 incidence in the UK beginning in February 2020 until August 2021. Data from https://ourworldindata.org/coronavirus#explore-the-global-situation

**Figure s20.** COVID-19 stringency index of the UK with indication of survey waves. Data from https://ourworldindata.org/coronavirus#explore-the-global-situation


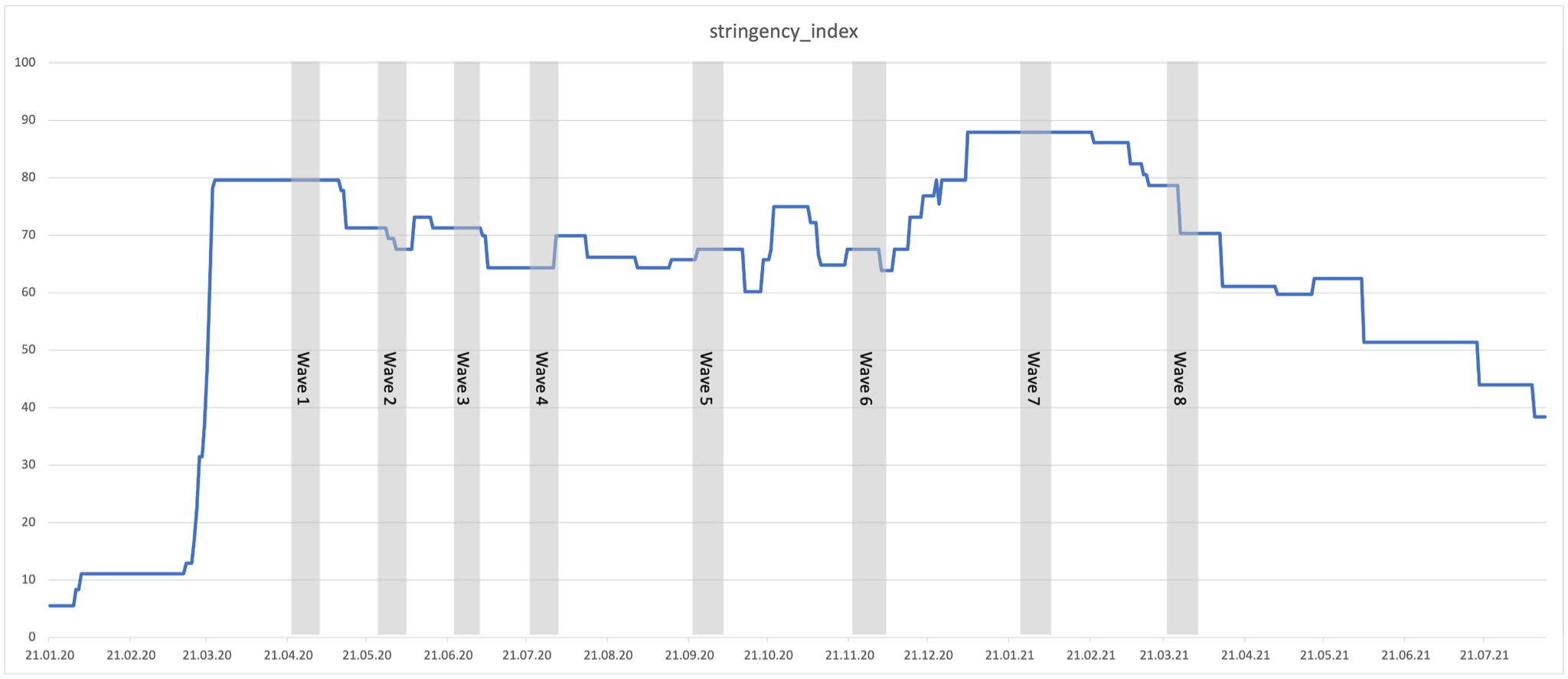

Supplement: Supplementary file 1 — Supplemental Material [file 41398_2023_2444_MOESM1_ESM.docx]
